# Reduced coupling between offline neural replay events and default mode network activation in schizophrenia

**DOI:** 10.1093/braincomms/fcad056

**Published:** 2023-03-03

**Authors:** Matthew M Nour, Yunzhe Liu, Cameron Higgins, Mark W Woolrich, Raymond J Dolan

**Affiliations:** Max Planck UCL Centre for Computational Psychiatry and Ageing Research, University College London, London WC1B 5EH, UK; Wellcome Trust Centre for Human Neuroimaging, University College London, London WC1N 3AR, UK; Department of Psychiatry, University of Oxford, Oxford OX3 7JX, UK; Wellcome Centre for Integrative Neuroimaging, University of Oxford, Oxford OX9 9DU, UK; Max Planck UCL Centre for Computational Psychiatry and Ageing Research, University College London, London WC1B 5EH, UK; State Key Laboratory of Cognitive Neuroscience and Learning, IDG/McGovern Institute for Brain Research, Beijing Normal University, Beijing 100875, China; Chinese Institute for Brain Research, Beijing 102206, China; Department of Psychiatry, University of Oxford, Oxford OX3 7JX, UK; Wellcome Centre for Integrative Neuroimaging, University of Oxford, Oxford OX9 9DU, UK; Department of Psychiatry, University of Oxford, Oxford OX3 7JX, UK; Wellcome Centre for Integrative Neuroimaging, University of Oxford, Oxford OX9 9DU, UK; Max Planck UCL Centre for Computational Psychiatry and Ageing Research, University College London, London WC1B 5EH, UK; Wellcome Trust Centre for Human Neuroimaging, University College London, London WC1N 3AR, UK; State Key Laboratory of Cognitive Neuroscience and Learning, IDG/McGovern Institute for Brain Research, Beijing Normal University, Beijing 100875, China

**Keywords:** schizophrenia, replay, decoding, resting state network, default mode network

## Abstract

Schizophrenia is characterized by an abnormal resting state and default mode network brain activity. However, despite intense study, the mechanisms linking default mode network dynamics to neural computation remain elusive. During rest, sequential hippocampal reactivations, known as ‘replay’, are played out within default mode network activation windows, highlighting a potential role of replay-default mode network coupling in memory consolidation and model-based mental simulation. Here, we test a hypothesis of reduced replay-default mode network coupling in schizophrenia, using magnetoencephalography and a non-spatial sequence learning task designed to elicit off-task (i.e. resting state) neural replay. Participants with a diagnosis of schizophrenia (*n* = 28, mean age 28.2 years, range 20–40, 6 females, 13 not taking antipsychotic medication) and non-clinical control participants (*n* = 29, mean age 28.1 years, range 18–45, 6 females, matched at group level for age, intelligence quotient, gender, years in education and working memory) underwent a magnetoencephalography scan both during task completion and during a post-task resting state session. We used neural decoding to infer the time course of default mode network activation (time-delay embedding hidden Markov model) and spontaneous neural replay (temporally delayed linear modelling) in resting state magnetoencephalography data. Using multiple regression, we then quantified the extent to which default mode network activation was uniquely predicted by replay events that recapitulated the learned task sequences (i.e. ‘task-relevant’ replay-default mode network coupling). In control participants, replay-default mode network coupling was augmented following sequence learning, an augmentation that was specific for replay of task-relevant (i.e. learned) state transitions. This task-relevant replay-default mode network coupling effect was significantly reduced in schizophrenia (*t*(52) = 3.93, *P* = 0.018). Task-relevant replay-default mode network coupling predicted memory maintenance of learned sequences (*ρ*(52) = 0.31, *P* = 0.02). Importantly, reduced task-relevant replay-default mode network coupling in schizophrenia was not explained by differential replay or altered default mode network dynamics between groups nor by reference to antipsychotic exposure. Finally, task-relevant replay-default mode network coupling during rest correlated with stimulus-evoked default mode network modulation as measured in a separate task session. In the context of a proposed functional role of replay-default mode network coupling, our findings shed light on the functional significance of default mode network abnormalities in schizophrenia and provide for a consilience between task-based and resting state default mode network findings in this disorder.

## Teaser

Resting brain activity exhibits spontaneous sequential neural reactivations (replay) and whole-brain network dynamics. Using a magnetoencephalography sequence learning task, Nour *et al.* show reduced temporal coupling between task-relevant neural replay and default mode network (DMN) activation in schizophrenia, during rest. Replay-DMN coupling related to memory and on-task DMN deactivation.

## Graphical Abstract

**Graphical Abstract:**
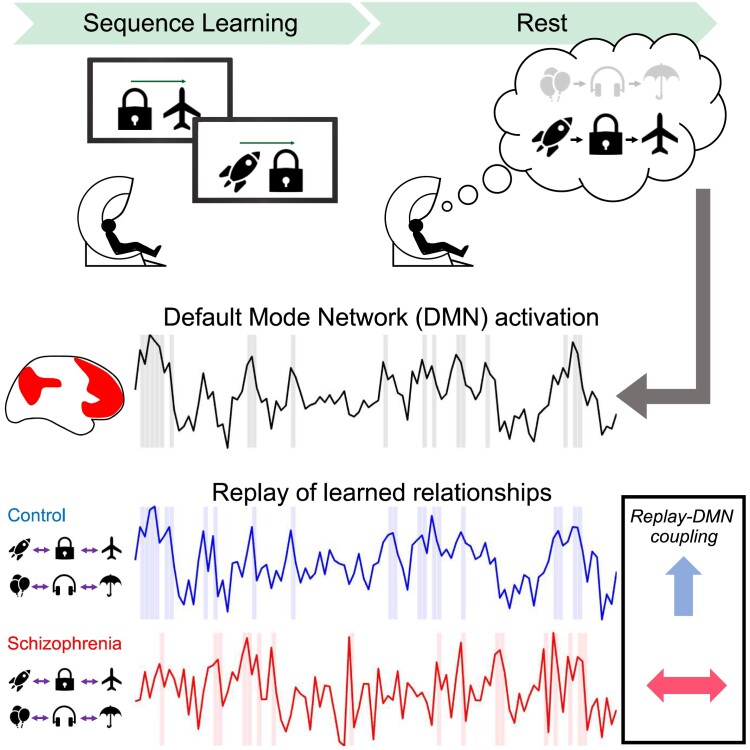

## Introduction

Humans spend much of the time disengaged from explicit goal-directed behaviour. During these (offline) ‘rest periods’, spontaneous brain activity transitions through attractor-like ‘resting state networks’ (RSNs), each exhibiting a distinct pattern of activity covariation across brain regions (i.e. functional connectivity).^1-5^ In psychiatry, the default mode network (DMN), a collection of (predominantly midline) brain regions exhibiting high resting metabolism and marked task-induced deactivations,^5,6^ has attracted particular attention.^7-15^ In schizophrenia—a debilitating neuropsychiatric condition with a lifetime prevalence approaching 1%^16^—DMN abnormalities include disturbances of intra- and inter-network functional connectivity during rest^8,9,13,17,18^ and attenuated DMN deactivation during task performance.^19-27^ However, despite over a decade of intense study, insight into the relationship between DMN dynamics and task-related cognition in schizophrenia is lacking owing to difficulties in indexing the representational content of brain activity measured at rest.^28,29^

Neural decoding provides a window into the role of offline brain activity in cognition,^28^ enabling investigators to track spontaneous reactivations of task-related neural activity patterns (‘neural representations’) during rest.^28^ Sequential reactivations during rest (or planning) periods have been shown to ‘replay’ learned associations between task states,^28,30-33^ in a manner thought to support memory consolidation, credit assignment and inference.^34-40^ Recent studies in humans and non-human primates suggest that replay events and DMN activation are temporally coordinated during rest.^41,42^ Replay-DMN coupling might provide a temporal window for offline updating of abstract internal representations (thought to be supported by DMN^43-46^) and allow a demarcation between task-evoked and offline neural computations.^41^ These functions appear pertinent to understanding the brain basis of psychotic symptoms, as in schizophrenia, which span aberrations of belief (e.g. paranoia) and internally generated perceptual experiences (e.g. hallucinations).

Replay signatures have been shown to be abnormal in people with a diagnosis of schizophrenia^47^ and genetic mouse models of the condition^48-50^ (e.g. impaired replay for inferred relationships and augmented replay-associated hippocampal ripple oscillations).Yet, these findings have not yet been related to previously reported disturbances of DMN activation in the condition. Here, we investigate replay-DMN coupling in schizophrenia using magnetoencephalography (MEG) data set and multivariate neural decoding. We relate this neural effect to behavioural measures of learning and abnormalities in task-evoked DMN deactivation. To anticipate our results, we show that schizophrenia is associated with impaired replay-DMN coupling during rest, specifically for replay events that mirror a learned task sequence (i.e. task-relevant replay events), indicating a potential role in memory consolidation. In line with this interpretation, replay-DMN coupling predicted mnemonic maintenance of task structural knowledge at the end of the scan.

## Materials and methods

### Data sets and participants

We availed of two existing MEG data sets. Data set A,^47^ which forms the focus of the primary replay-DMN analyses, comprises MEG data (see below) from 28 people with a diagnosis of schizophrenia (mean age 28.2 years, range 20–40, 6 females, diagnosis assessed with the Structured Clinical Interview for DSM-IV-TR, Axis I Disorders, SCID-I^51^) recruited from London community psychosis NHS clinics and 29 healthy volunteers (mean age 28.1 years, range 18–45, 6 females) recruited from the same geographical area through online advertisements. Thirteen patients were not taking dopamine 2/3 receptor (D2/3R) antagonist medication (one medication naïve). Groups were matched for age, gender, IQ and educational attainment (see Supplementary Table 1 for demographics and Supplementary Materials and Methods for exclusion criteria and clinical assessment). Data set B,^4^ used to train the hidden Markov model (HMM) RSN observation models, comprises 5-min resting state MEG scans and structural MRI scans from 55 healthy volunteer participants (mean age 26.5 years, range 18–48, 20 females). Studies were approved by the London Westminster NHS Research Ethics Committee (15/LO/1361) and University of Nottingham Medical School Research Ethics Committee. Participants’ consent was obtained according to the Declaration of Helsinki.

### Replay experimental protocol (Data set A)

Participants completed three identical sessions of an applied learning task during MEG scanning, in which they needed to infer how eight task pictures formed two sequences ([A → B → C → D] and [A’ → B’ → C’ → D’], termed ‘structural sequences’). This task has previously been shown to elicit spontaneous neural replay of learned sequential relationships during a post-learning resting session.^30,47^ During each session, participants passively observed three unique ‘visual sequences’ (four times each). Each ‘visual sequence’ contained four task pictures that were presented in a ‘scrambled’ order relative to the underlying ‘structural sequences’ (e.g. [C’ → D’ → C → D], [B → C → B’ → C’] and [A’ → B’ → A → B]). Prior to the scan, participants were explicitly taught an ‘unscrambling rule’ that described in full the relationship between these scrambled (‘visual’) sequences and the underlying (‘structural’) sequences. This rule stated that only the first and last transition in each ‘visual sequence’ reflected a true transition in a ‘structural sequence’. During MEG, when presented with a new set of stimuli, participants could use this knowledge to infer the correct ‘structural’ relationships from the presented scrambled ‘visual sequences’. They were not required to respond in any way while viewing the ‘visual sequences’. After three applied learning sessions, participants completed a 5-min ‘post-learning’ MEG resting state scan (‘POST’, eyes open). Participants completed a 12-question ‘quiz’ after each applied learning session, and immediately following the post-learning rest session. This enabled us to confirm that patients and controls had equivalent knowledge of structural sequences after three sessions of applied learning (‘Quiz 3’, immediately prior to the post-learning rest) and immediately after post-learning rest (‘post-rest quiz’) (patients showed learning impairments after the first and second applied learning sessions relative to controls; for full behavioural results, see Nour *et al.*^47^).

**Figure 1:**
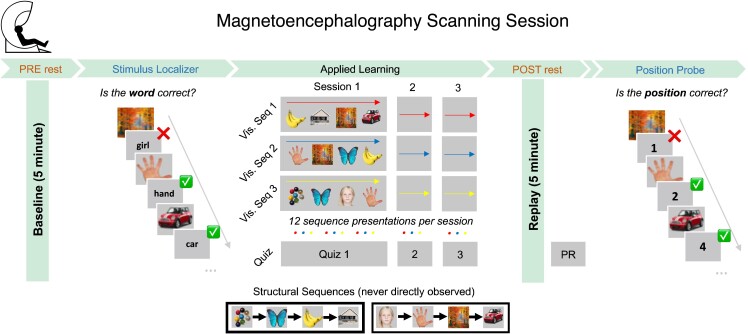
**MEG task outline.** The MEG session began with a 5-min eyes open (pre-learning) rest session and a stimulus localizer. Participants then completed three applied learning sessions (∼5 min each), in which they were shown ‘visual sequences’ containing eight task pictures in a scrambled order and needed to infer the correct sequential relationships between them (i.e. task ‘structural sequences’) using an unscrambling rule learned during a pre-scan training visit. Applied learning was followed by a second 5-min eyes open (post-learning) rest session. Structural knowledge was assessed in several quiz sessions [Q1, Q2, Q3 and post-rest (PR)]. The scan session finished with a position probe task to assess knowledge maintenance. See ‘Materials and Methods’ section for full details, and Nour *et al*.,^47^ for behavioral and replay findings.

Prior to the applied learning MEG sessions, participants completed a ‘pre-learning’ MEG resting state session (‘PRE’, 5 min, eyes open) and a stimulus localizer task (to gather visually evoked neural data used to train neural classifiers, below). At the end of the scanning session, participants completed a final position probe task to assess their knowledge of the abstract task structure (i.e. which picture occupied which ordinal position in the ‘structural sequence’). See Fig. 1 for MEG task schematic and Supplementary Materials and Methods for further details).

### MEG acquisition and preprocessing

In both Data sets A and B, MEG was recorded continuously at 1200 samples/s using a whole-head 275-channel axial gradiometer system (CTF Omega, VSM MedTech). Preprocessing pipelines for Data sets A and B were identical to our previous healthy volunteer replay RSN report^41^ (see Supplementary Materials and Methods for further details).

### Replay analysis pipeline

**Figure 2:**
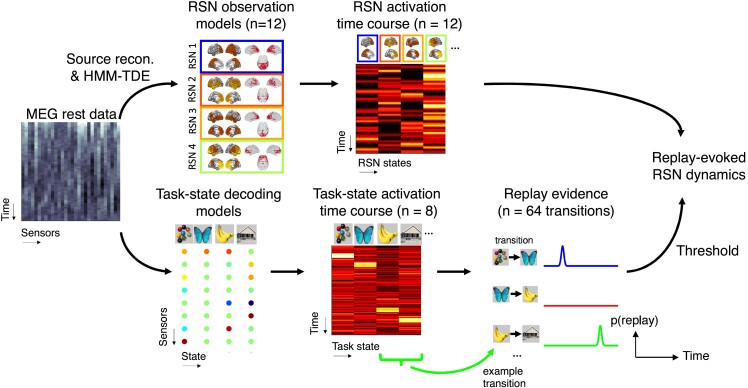
**Decoding spontaneous replay and DMN activations during rest. ‘**Dual decoding’ analysis pipeline. We decoded two families of activation time courses from MEG data of each participant: the first is a family of resting state network (RSN) state activation time courses (top, total *n* = 12, 4 shown only) on MEG data in source space, using a hidden Markov model time-delay embedding (TDE-HMM) framework,^4,41,52^ and the second is a family of task state reactivation time courses (bottom, total *n* = 8, 4 shown only), in sensor space, using a temporally delayed linear modelling (TDLM) framework.^33,47^ See ‘Materials and Methods’ section for details.

Replay detection analysis was conducted in sensor space. Readers are directed to our previous report for full details.^47^ Briefly, for each participant, we trained a separate sparse logistic regression classifier for each MEG task picture, using labelled MEG sensor data from stimulus localizer (a separate family of classifiers trained at each time point of the stimulus-evoked response). We identified the time point of peak decodability at the group level in cross validation (180 ms after stimulus onset), and for each participant, we applied these decoders to MEG rest data (pre- and post-learning). This enables us to derive a state reactivation time series for each task picture (e.g. reactivation time course of picture $A=\;\sigma (X\beta_{A})$, where *X* is the [time, sensors + 1] matrix of MEG data, $\beta_{A}$ is the [sensors + 1, 1] trained classifier for State A, and σ(⋅) is the logistic sigmoid transform). We used the state-specific reactivation time series to generate a ‘replay probability’ time course for each unique state → state replay transition (e.g. 64 in total reflecting 8 ∗ 8 possible state pairs). For example, for transition [A → B] we computed the element-wise product of the reactivation time course of State A and time-lagged reactivation time course of State B (i.e. $R_{t}^{\left[A\to B\right]}=\;A_{t}*B_{t+\tau }$, where τ = 40 ms, corresponding to the time lag of peak replay detected in the present data set^47^ and earlier reports in healthy volunteers^30,31^). We thresholded this probabilistic ‘replay evidence’ output at the transition-specific 99th percentile^41^ to provide a time course of ‘replay onsets’ used throughout this paper (see Fig. 2 and Supplementary Materials and Methods for further details).

### RSN modelling

The RSN analysis pipeline was conducted in source space (see Supplementary Materials and Methods). We use an established time-delay embedding HMM (TDE-HMM) approach to RSN analysis to infer the activation time courses of each RSN for each participant and session separately.^4,41,52^ Briefly, the approach assumes that at each time point, *t*, the brain is in one of *K* ‘latent states’ (each a distinct RSN), which are parameterized as probability distributions over observable MEG data features that capture each RSN’s functional connectivity pattern (i.e. ‘RSN observation models’), and where the latent state → state transition probabilities respect a first-order Markovian property. See Supplementary Materials and Methods for mathematical details, including model fitting.

In the present work, we use a two-step approach to RSN activation time course inference, as in Higgins *et al*.^41^ We first fitted the complete TDE-HMM to Data set B (*n* = 55 healthy control participants), to infer the probabilistic observation models for each RSN (corresponding to the RSN functional connectivity patterns). As in Higgins *et al.*,^41^ we set *K* to 12 and identify RSN2 as the DMN owing to high-power in medial frontal and temporal regions and coherent oscillations in the lateral parietal cortex (Supplementary Fig. 1 for fitted observation models for each RSN). We then applied the fitted observation models (one for each of 12 RSNs) to MEG data from Data set A (clinical data set), to infer participant-specific RSN activation time courses and latent state transition probabilities. This two-step procedure enables a direct comparison between our results and previously published MEG results while also affording greater confidence with respect to the anatomical distributions of activity for each RSN, owing to availability of high-resolution MRI used for MEG co-registration in Data set B.^4^

The HMM procedure yields a [time, RSN] activation time course matrix for each participant and session in Data set A. Prior to replay- (and stimulus-) evoked RSN activation analysis, we first mean-centred the columns of this RSN activation matrix for each participant and session.^41^ We found no significant differences between patients and controls in dynamical or spectral properties of inferred RSN state activations (see Fig. 2, Supplementary Fig. 2 and Supplementary Results).

### Replay-evoked RSN activation

To investigate if RSN activation dynamics were modulated by spontaneous replay events, we epoched the [time, RSN] activation probability matrix from −500 to +500 ms with respect to identified replay onsets. We performed this epoching procedure separately for replay events comprising each of 64 unique state → state transitions (all ordered pairs of eight task states). Replay event ‘onset’ was defined as a time point exhibiting replay evidence exceeding the transition- and session-specific 99th percentile^41^ (see Supplementary Materials and Methods for further details of replay epoching procedure). We found no significant difference in number of included ‘replay onsets’ between patient or control groups for any replay transition (see Supplementary Results**)**. We then generated a single [time, RSN] matrix for each replay transition (*n* = 64) by averaging the replay-evoked activation profile over all included replay events for each transition separately. We repeated this procedure for each participant and rest session (pre- and post-learning) separately.

This transition-specific epoching procedure was critical in allowing us to examine whether replay-DMN coupling was modulated by replay content (i.e. which state pair was replayed, see Supplementary Materials and Methods for further discussion). Our primary question was whether replay RSN coupling was greater for replay events containing ‘task relevant’ state pairs (i.e. replay events where the two reactivated states were adjacent in the learned ‘structural sequences’), compared to that for a coupling averaged over all possible state → state transitions (*n* = 64, a ‘non-specific’ effect). We formally addressed this using multiple regression. Specifically, for each peri-replay time point (−500 to +500 ms), RSN, rest session and participant, we regressed the [64, 1] vector of RSN activation probabilities (one entry for each replay transition, e.g. [A → B], [B → C], …) onto a design matrix comprising a regressor for correctly inferred (i.e. ‘structurally adjacent’ or ‘task relevant’) transitions and a constant term that models a non-specific (baseline) replay RSN association. When applied to the DMN activation time course, this yields regression coefficient estimates for both the task-relevant replay effects of interest (*β*_inferred[DMN]_) and background non-specific effects (*β*_non-specific[DMN]_) at every peri-replay time point. Contrasting these effects from pre-learning rest to post-learning rest yields an estimate of the boosting of such task-relevant replay RSN coupling after learning (Δ*β*_inferred[DMN]_ = *β*_inferred[DMN][POST]_ − *β*_inferred[DMN][PRE]_). See Supplementary Materials and Methods for further details.

**Figure 3:**
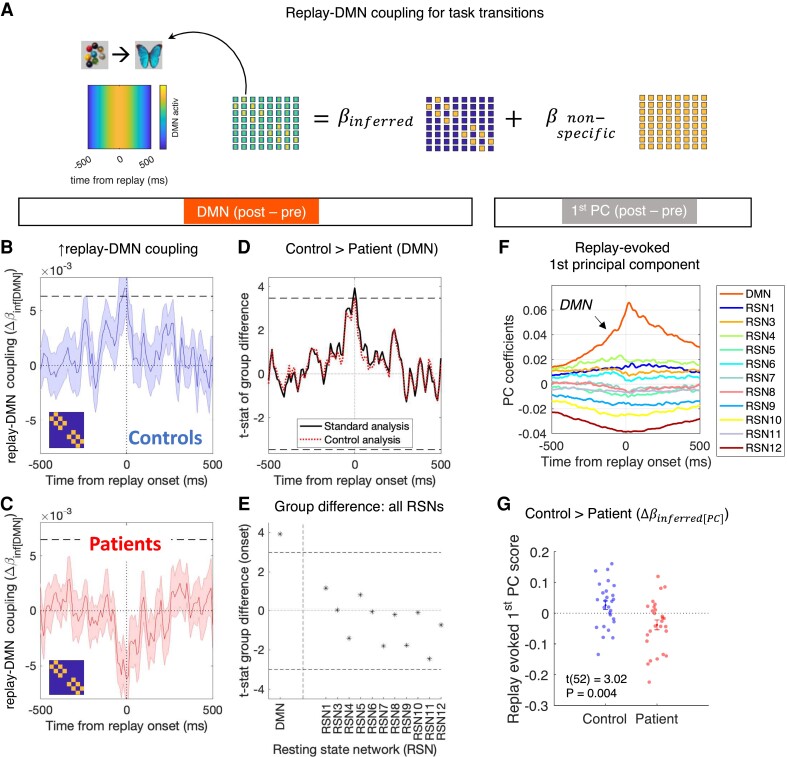
**Patients show a reduced replay-default mode network (DMN) coupling specific for inferred transitions.** (**A**) For each participant, we calculated the mean DMN activation time course ±500 ms around replay onset for each unique state → state transition (8 ∗ 8 = 64 unique pairwise transitions. Illustrative transition magnified on left). For each participant and peri-replay time-point in turn, we then regressed a [64, 1] vector of DMN activation probabilities (one entry for each replay transition, e.g. [A → B], [B → C], …) onto a design matrix comprising a regressor for correctly inferred (i.e. task-relevant ‘structurally adjacent’) transitions (*β*_inferred_ regressor) and a constant term that models a baseline (non-specific) replay-DMN association (*β*_non-specific_). (**B**) Mean ± SEM (over control participants) change in replay-DMN coupling for inferred (structurally adjacent) transitions (inset) from pre- to post-learning rest (Δ*β*_inferred[DMN]_). Control participants show a significant increase in replay-DMN coupling at replay onset at *P*_FWE_ < 0.05 (horizontal dashed line denotes peak-level *P*_FWE_ = 0.05, right-tailed, derived from subject sign flip permutation test, 500 permutations, controlling for multiple comparisons from −500 to +500 ms from replay onset). For similar results in pre- and post-learning MEG rest data alone, see Supplementary Fig. 4. (**C**) Mean ± SEM (over patient participants) change in replay-DMN coupling for inferred transitions (inset) from pre- to post-learning rest, as in (**B**). Patient participants show no significant increase in Δ*β*_inferred[DMN]_ at any time point (right-tailed *P*_FWE_ < 0.05 significance threshold, as in **B**). Of note, the negative deflection observed at 0 ms is also non-significant, under a two-sided hypothesis (*P*_FWE_ = 0.15). (**D**) A formal statistical comparison of group differences (control > patient) for the Δ*β*_inferred[DMN]_ effect in (**B**) and (**C**), showing a maximal effect at replay onset time (solid black line shows *t*-statistic from two-sample two-tailed *t*-test at each time point). This effect exceeded peak-level *P*_FWE_ < 0.05, two-tailed (threshold depicted by horizontal dashed lines, derived from a non-parametric group-membership permutation test, 500 permutations, controlling for multiple comparisons from −500 to +500 ms from replay onset). Red dashed line shows the group difference effect calculated after first regressing out any variance in replay-DMN coupling that is attributable to the replay strength *per se* at each time point separately (see main text). (**E**) We repeated the above analysis for all other RSNs and here plot the *t*-value of group differences in Δ*β*_inferred_$\Delta \beta_{inferred}$ at replay onset (0 ms) for each RSN separately. Horizontal dashed line depicts statistical significance threshold for two-sample two-tailed *t*-test at *P* < 0.05, Bonferroni corrected for multiple comparisons over RSN states. (**F**) Top: coefficients of the first principal component (PC) of the replay-evoked RSN activation time courses (see ‘Materials and Methods’ section and Supplementary Fig. 5). (**G**) Mean ± SEM (over participants) of the Δ*β*_inferred[PC]_ effect, calculated using a multiple regression approach (as in **A**), but now using the first PC score of the replay-evoked response (as in **F**) as the dependent variable. Sample for all analyses: patients *n* = 27, controls *n* = 27.

We also used a complementary approach to examine global patterns of replay-evoked RSN dynamics that span several time points and RSNs. Using principal component analysis (PCA), we projected the replay-evoked RSN activation data (for each participant, an [event, time ∗ RSN] matrix of evoked RSN activations, where ‘events’ are *n* = 64 individual replay transitions, and the [time, RSN] data corresponding to each ‘event’ has been vectorized into a single dimension) onto new principal component (PC) axes (yielding an [event, PC] data matrix). Here, each PC axis is defined as a [time, RSN] coefficient matrix capturing an orthogonal axis of variation in the observed data. The first PC, which captures the ‘principal axis of variation’ in the observed MEG data, demonstrates an expected ‘DMN activation’ profile (see Fig. 3F and Supplementary Fig. 5). For each participant, we can then quantify the expression of this ‘DMN activation’ profile as a single number for each replay transition (first PC score) and identify the extent to which this expression profile is uniquely associated with task-relevant replay events using multiple regression, as above (task-relevant replay coefficient denoted *β*_inferred[PC]_). To ensure that our projection from native to PC space is identical for all participants and across both pre- and post-learning rest sessions, we conduct the PCA on the evoked neural data concatenated over all participants and rest sessions (see Supplementary Materials and Methods for complete details).

### Stimulus-evoked RSN activation

**Figure 4:**
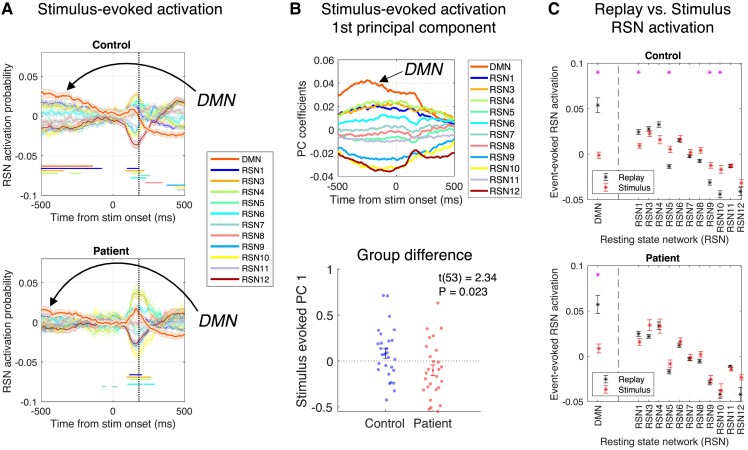
**Patients exhibit reduced stimulus-evoked RSN modulation.** (**A**) Mean ± SEM (over participants) of stimulus-evoked RSN activation dynamics, using MEG data from stimulus-evoked response in stimulus localizer (prior to learning); 0 ms denotes stimulus onset [negative times indicate pre-stimulus inter-trial interval (ITI)]. Vertical dashed line denotes time slice from which training data were taken for task state decoders (180 ms). * denotes *P*_FWE_ < 0.05 significant activation time clusters (see Supplementary Materials and Methods). Effects for each participant were calculated as the mean effect over all trials in stimulus localizer (note, as this task was completed prior to the learning task, evoked neural responses were devoid of any information pertaining to underlying task structure). Patients *n* = 27, controls *n* = 28. (**B**) Top: coefficients of the first PC in the stimulus-evoked RSN activation time course (shown in **A**). PCA aligned to that performed in rest data, shown in Fig. 3. See also Supplementary Fig. 5). Bottom: reduced expression of this stimulus-evoked default mode network (DMN) deactivation pattern in patients (mean ± SEM over participants, where each participant’s value is the mean of the first PC score over all trials of the stimulus localizer). Patients *n* = 27, controls *n* = 28. (**C**) Mean ± SEM (over participants) of replay-evoked (black, from 0-ms time sample) and stimulus-evoked (red, from 180-ms time sample) effect for each RSN. The replay-DMN association (averaged over all possible task state transitions) is significantly greater than the association attributable to a similarity between the DMN observation model and the stimulus-evoked response at the time of decoder training (shown in **A**). This rules out a possibility that the replay-DMN association detected during rest might trivially arise out of a similarity between visually evoked neural data (used for task state decoder training) and the DMN observation model.^41^ * denotes statistical significance (Wilcoxon signed rank test for equal medians, paired samples, two-tailed, *P* < 0.05, Bonferroni corrected for multiple comparisons across RSNs). Patients *n* = 27, controls *n* = 27.

Finally, we use MEG data from the Stimulus Localizer task to examine stimulus-evoked RSN activation dynamics. Here, we epoched the [time, RSN] activation probability matrix from −500 to +500 ms with respect stimulus onset in each trial (time points before 0 ms represent a grey screen inter-trial interval period) and used a similar PCA approach to identify the axis of maximal variation in this stimulus-evoked RSN activation dynamics (here the first PC reveals a ‘DMN deactivation’ profile, Fig. 4B) (see Supplementary Materials and Methods).

### Statistical analysis and software

We control for multiple statistical comparisons throughout and consider family-wise error corrected *P* < 0.05 (two-tailed) as statistically significant, unless otherwise stated. Specifically, we used non-parametric permutation tests to assess the statistical significance of effects where we wished to control for multiple comparisons across time points (i.e. evoked RSN dynamics) or frequencies (i.e. RSN spectral properties) (see Supplementary Materials and Methods for mathematical details). When considering single-variable effects or bivariate correlations, we conducted a formal test that the effects in question were sampled from a population with a normal distribution (Shapiro–Wilk test) prior to using parametric tests (e.g. unpaired *t*-test and Pearson’s correlation) and used non-parametric equivalent tests where this null hypothesis was rejected at *α* = 0.05 (e.g. Wilcoxon rank sum test for equal medians, correlation and regression analyses conducted on rank ordered variables). For between-subjects multiple regression analyses, ‘group’ was effects coded (patients = −0.5, controls = +0.5) unless otherwise stated. For all analyses, summary effects are reported as mean ± 1 standard error of the mean (SEM), and two-tailed *P* < 0.05 is deemed significant, unless otherwise stated.

## Results

### Decoding spontaneous replay and DMN activations during rest

Participants completed a sequence learning task during MEG, followed by a 5-minute resting state scan (Fig. 1). As described in our prior work,^30,47^ this protocol elicits spontaneous neural replay of inferred sequential relationships during a post-learning rest session, detected using a multivariate neural decoding (see ‘Materials and Methods’ section).^33^ Here, we focus on a whether spontaneous replay events during rest co-occur with RSN activations, focusing on DMN. We first use a ‘dual decoding’ approach to identify sequential task state reactivations (replay) and DMN activation in MEG rest data (see ‘Materials and Methods’ section and Fig. 2). We then test whether DMN activation is uniquely predicted by replay events containing task-relevant (‘inferred’) state transitions (i.e. events that replay adjacency relationships in the task ‘structural sequences’) using a multiple regression approach (see ‘Materials and Methods’ and Fig. 3A).

### Patients show reduced replay-DMN coupling specific for inferred transitions

Control participants alone exhibited a significant increase in the degree to which DMN activation was predicted by onset of task-relevant replay events (i.e. ‘task-relevant’ replay-DMN coupling), from pre- to post-learning rest sessions (controls Δ*β*_inferred[DMN]_ = 0.007 ± 0.002, peak-level *P*_FWE_ = 0.036, patients: Δ*β*_inferred[DMN]_ = −0.006 ± 0.002, peak-level *P*_FWE_ = 1.00, non-parametric sign-flip permutation test, right-tailed, effects at 0 ms, controlling for multiple comparisons over time, Fig. 3B and C). ‘Task relevant’ replay-DMN coupling was significantly greater in control participants compared to patients (*t*(52) = 3.93, peak-level *P*_FWE_ = 0.018, non-parametric group-membership permutation test, two-tailed, effect at 0 ms, controlling for multiple comparisons over time, Fig. 3D). For similar results in post-learning MEG rest data alone, see Supplementary Fig. 4. As expected, we found no significant task-relevant replay-DMN coupling during pre-learning rest (Supplementary Fig. 4).

The group difference in task-relevant replay-DMN coupling (Δ*β*_inferred[DMN]_) was not a consequence of impaired neural replay in the patient group *per se*.^47^ First, we found no correlation between Δ*β*_inferred[DMN]_ (at replay onset) and replay strength across participants (*r*(52) = 0.19, *P* = 0.17, Pearson’s correlation, ‘replay strength’ defined as the increase in ‘sequenceness’, at 40-ms replay lag, from pre- to post-learning rest sessions, reported in Nour *et al.*^47^). Second, the group difference in Δ*β*_inferred[DMN]_ remained significant after controlling for variance attributable to replay strength using multiple regression (Fig. 3D). Furthermore, a group difference in Δ*β*_inferred[DMN]_ was not a trivial consequence of impoverished knowledge of task structure during the post-learning rest session, as both groups exhibited ceiling level knowledge on an explicit knowledge quiz both immediately prior (quiz after final learning session (Q3): controls = 99.4% ± 0.43, patients = 97.8% ± 0.72) and immediately after the rest session [post-rest (PR) quiz: controls = 99.1% ± 0.51, patients = 98.8% ± 0.58; see Fig. 1 for quiz timing]. The group difference in Δ*β*_inferred_ was not significant for any other RSN at replay onset (Fig. 3E).

We found a similar pattern of results using a complementary PCA approach, which enables us to quantify replay-evoked ensemble activation patterns across time points and RSNs for each participant and replay transition using a single number (‘Materials and Methods’ section). The first PC pattern revealed a ‘DMN-dominant’ profile (Fig. 3F). The specificity of this ‘DMN-dominant’ first PC for task-relevant replay events was again greater in controls compared to patients (Δ*β*_inferred[PC]_ in controls = 0.026 ± 0.014, in patients = −0.038 ± 0.016, group difference: *t*(52) = −3.02, *P* = 0.004, two-sample two-tailed *t*-test, Fig. 3G). Intriguingly, patients showed a negative Δ*β*_inferred[PC]_ effect, indicating not only that, after learning, DMN activation fails to show a boosted coupling to task-relevant replay events (compared to non-specific background), but also that in some participants, such coupling is reduced. Examining the Δ*β*_inferred[PC]_ effect during post-learning rest in isolation revealed a significant effect in control participants alone. Δ*β*_inferred[PC]_ was not significantly different from 0 in either group during pre-learning rest (see Supplementary Results).

Importantly, variance in Δ*β*_inferred[PC]_ was not simply a reflection of replay strength *per se* (Pearson’s correlation between Δ*β*_inferred[PC]_ and replay strength across participants: *r*(52) = 0.03, *P* = 0.82). Moreover, the group difference in Δ*β*_inferred[PC]_ remained significant after controlling for D2/3R antagonist (antipsychotic) medication exposure (regressing Δ*β*_inferred[PC]_ on diagnosis and chlorpromazine-equivalent daily dose^53^ revealed a significant main effect of diagnosis (*β*_diagnosis_ = −0.052 ± 0.025, *t* = −2.13, *P* = 0.038) but not medication (*β*_CPZ_dose_ = −0.0003 ± 0.0004, *t* = −0.94, *P* = 0.35), where ‘diagnosis’ coded as control = 0, patient = 1).

These findings are consistent with an impairment in replay-DMN coupling for inferred transitions in patients and rule out a possibility that this is a trivial consequence of a failure to learn the task sequences, impaired replay *per se* or medication exposure. We additionally show that a replay-DMN coupling, as previously shown in control participants,^41^ has a specificity for replay of events corresponding to (task-relevant) transitions that reflect an inferred task structure.

### Patients exhibit reduced stimulus-evoked RSN modulation

We next asked whether patients also exhibited differences in stimulus-evoked RSN dynamics during task conditions and whether this on-task (online) effect is related to resting (offline) RSN dynamics. A comparison between online and offline RSN dynamics is rendered possible as we use the same RSN observation models to infer RSN activation time courses in rest and task data (as in Higgins *et al.*^41^).

In visually evoked MEG data from stimulus localizer, we found no significant DMN activation either at stimulus onset (0 ms) nor at the time point from which we subsequently extracted MEG training data for neural state decoders (time of peak stimulus decodability, 180 ms), in either patients or controls. Instead, we observed a pattern of relative DMN activation during an inter-trial interval (a stimulus-free ‘rest’ period) and relative DMN deactivation ∼200 ms after stimulus onset (Fig. 4A). This ‘DMN deactivation’ pattern was also reflected in the coefficients of the first PC derived from a PCA analysis on stimulus-evoked RSN activation time courses (see ‘Materials and Methods’ section). Notably, the stimulus-evoked RSN ensemble response, as projected onto this first PC, was significantly more pronounced in controls relative to patients with schizophrenia (mean first PC scores over all trials in controls = 0.82 ± 0.05, patients = −0.10 ± 0.6, *t*(53) = 2.34, *P* = 0.02, two-sample *t*-test, two-tailed, Fig. 4B). This is consistent with functional MRI (fMRI) reports of attenuated DMN suppression in schizophrenia during task performance.^19-27^

A group difference in stimulus-evoked RSN modulation is unlikely to be explained by differential attention during the stimulus localizer, as all participants achieved over 90% accuracy during an incidental task of attention and semantic processing (see Fig. 1 and ‘Materials and Methods’ section), with no group difference (patient accuracy = 98.5% ± 0.30, control accuracy = 98.3% ± 0.33%, *z*(53) = −0.62, *P* = 0.54, two-sample Wilcoxon rank sum test for equal medians, two-tailed) nor any correlation between task performance and first PC score across participants (*ρ*(53) = −0.12, *P* = 0.40, Spearman’s correlation). Moreover, the group difference in the stimulus-evoked (DMN deactivation) first PC score remained after controlling for D2/3 antagonist (antipsychotic) medication exposure, using a similar multiple regression approach as in the replay-evoked analysis (*β*_diagnosis_ = −0.23 ± 0.091, *t* = −2.56, *P* = 0.013, *β*_CPZ_dose_ = 0.001 ± 0.001, *t* = 1.08, *P* = 0.29). Of note, a similar group difference in stimulus-evoked DMN deactivation is found when using an alternative measure to that derived from PCA (control = 0.030 ± 0.005, patient = 0.012 ± 0.005, *z*(53) = 2.72, *P* = 0.007, two-sample Wilcoxon rank sum test for equal medians, two-tailed, here defining DMN deactivation as the difference between mean DMN activation measured in pre-stimulus (−500 to 0 ms) and post-stimulus (0–500 ms) epochs).

### Relationship between replay-evoked and stimulus-evoked RSN dynamics

**Figure 5:**
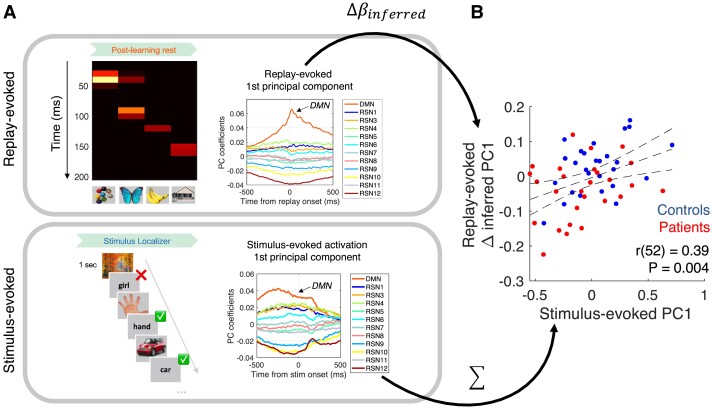
**Relationship between replay-evoked and stimulus-evoked RSN dynamics.** (**A**) We extracted the principal mode of variation governing evoked RSN dynamics for both offline (replay-evoked, top) and online (stimulus-evoked, bottom) conditions using PCA (see ‘Materials and Methods’ section). The stimulus-evoked response is defined as the mean of the first PC score over all trials of stimulus localizer, while for replay-evoked response, we quantify the degree to which the first PC score is expressed specifically for inferred transitions, and increases from pre- to post-learning (i.e. Δ*β*_inferred[PC]_). PC coefficients duplicated from Figs 3F and 4B. Illustrations on left: top is an illustration of an exemplary spontaneous replay event in the [time, state] decoding matrix of a single control participant. Bottom is a depiction of the stimulus localizer trial structure [a blank screen inter-trial interval (ITI) precedes each picture onset, not shown]. (**B**) Positive linear correlation between stimulus-evoked RSN-modulation and replay-evoked RSN modulation (Δ*β*_inferred[PC]_, increase from pre- to post-learning specific for inferred transitions). Variable definition is the participant-specific first PC scores, as in (**A**). Although the mean effect of both measures is lower in patients compared to controls (see Figs 3 and 4), the linear relationship between variables is preserved across participants after accounting for these group differences in mean effects (see main text). Error bars represent 95% confidence intervals of line of best fit. Sample: *n* = 27 patients and *n* = 27 controls.

An observation that patients exhibit attenuated DMN modulation, both with respect to external stimuli (online) and spontaneous replay events (offline), provides a unique opportunity to investigate the relationship between these two facets of evoked DMN dynamics. Consequently, we examined the correlation between each participant’s stimulus-evoked first PC score (mean over trials for each participant) and replay-evoked first PC score (defined as Δ*β*_inferred[PC]_, the increase in replay-DMN coupling for inferred transitions, from pre- to post-learning rest). This revealed a positive relationship across all participants (*r*(52) = 0.39, *P* = 0.004, Pearson’s correlation, Fig. 5), which remained significant when controlling for a difference in Δ*β*_inferred[PC]_ between groups (multiple regression of replay-evoked Δ*β*_inferred[PC]_ effect onto stimulus-evoked first PC score, group membership and the interaction of these variables: *β*_stim-evoked_ = 0.097 ± 0.037, *t*(50) = 2.58, *P* = 0.013, *β*_group_ = 0.052 ± 0.021, *t*(50) = 2.48, *P* = 0.016, *β*_stim-evoked∗group_ = 0.051 ± 0.075, *t*(50) = 0.68, *P* = 0.50, with equivalent statistical results when switching the assignment of dependent and independent MEG variables in regression). The non-significant interaction term in this regression suggests a linear relationship between online and offline RSN modulation reflects a fundamental aspect of brain functional network organization that is preserved in schizophrenia. The relationship between stimulus-evoked and replay-evoked DMN dynamics was also present when using direct measures of DMN activation that do not make use of PCA (*ρ*(52) = 0.42, *P* = 0.002, Spearman’s correlation, with replay-evoked DMN activation defined as the mean Δ*β*_inferred[DMN]_ effect from −100 to +100 ms with respect to replay onset, and stimulus-evoked DMN deactivation defined as the reduction in mean DMN activation from pre-stimulus to post-stimulus 500-ms epochs).

### Relationship to behaviour

Having found group differences in replay evoked DMN modulation, we next asked whether subject-level neural effects predict maintenance of structural knowledge, which we examined in a position probe quiz at the end of the scanning session (Fig. 1 and ‘Materials and Methods’ section). Patients performed numerically (although not significantly) worse on this behavioural measure compared to controls (controls = 96.6% ± 0.75, patients = 93.7% ± 1.72, *z*(52) = 1.23, *P* = 0.22, Wilcoxon rank sum test for equal medians, two-tailed). Across all participants, performance positively related to the degree to which DMN activation is specifically coupled to replay of inferred transitions during the post-learning rest period (*β*_inferred[PC]_, *ρ*(52) = 0.31, *P* = 0.02, Spearman’s correlation), and this remained significant after controlling for a group difference in mean performance (multiple regression of performance onto *β*_inferred[PC]_, group and their interaction, using ranked variables: *β*_replay-evoked_ = 0.31 ± 0.14, *t*(50) = 2.24, *P* = 0.03, *β*_group_ = 2.71 ± 4.29, *t*(50) = 0.63, *P* = 0.53, *β*_replay-evoked∗group_ = 0.22 ± 0.28, *t*(50) = 0.79, *P* = 0.43). Of note, there was no relationship between replay-DMN coupling for inferred transitions (*β*_inferred[PC]_) and speed of knowledge acquisition itself (‘learning lag’, *ρ*(52) = −0.09, *P* = 0.52, Spearman’s correlation), a behavioural measure we previously showed was related to replay strength.^47^

Next, we investigated the relationship between stimulus-evoked RSN responses and task performance during stimulus localizer itself (Fig. 1 and ‘Materials and Methods’ section). We found greater expression of a stimulus-evoked ‘DMN deactivation’ profile predicted faster response reaction time (RT) in the stimulus localizer task at the single trial level, with no group difference in this MEG–behaviour relationship (results from a single multiple regression concatenating all correctly answered trials across participants, and regressing trial RT onto a design matrix comprising stimulus-evoked first PC score (main effect), a group × PC score interaction term, and nuisance regressors controlling for participant-specific mean RT for congruent and incongruent trials separately: *β*_PC_score_ = −1.95 ± 0.61, *t*(19 838) = −3.19, *P* = 0.001. *β*_group×PC_score_ = −0.63 ± 1.22, *t*(19 838) = −0.51, *P* = 0.61.

### Relationship to symptoms

Finally, we found no relationship between the expression of positive psychotic symptoms or depressive symptoms and either replay-evoked DMN modulation or stimulus-evoked DMN modulation in our patient sample. Negative symptoms showed significant associations with post-learning replay-evoked DMN modulation (*β*_inferred[PC]_) and stimulus-evoked DMN modulation, but these did not survive a correction for multiple statistical comparisons (Supplementary Table 2). Neural effects were not different between patients taking and not taking D2/3R antagonist medication (Supplementary Table 2).

## Discussion

DMN function has been construed in terms of internally directed and self-referential thought.^9,54-58^ However, more recent proposals suggest a role in abstract cognitive functions that involve domain-general representations of the world (i.e. internal models or schemas),^43-46^ a function of immediate relevance for understanding myriad psychiatric symptoms such as delusions. These representations (cognitive maps) are supported by circuits that sit at the apex of a cortical processing hierarchy (including hippocampus), one suggested as enabling relational inferences that ‘go beyond’ direct experience.^44,59^ Offline hippocampal replay is intimately connected to these functions. Replay trajectories are thought to reflect a sampling from internal relational models^30,39,40^ that in turn support model extension and consolidation.^34,35,60-63^ One hypothesis is that a coupling between DMN activation and hippocampal replay^41^ (and associated ripple oscillations^42^) during rest could provide a temporal processing window that enables modification of, and sampling from, internal models, in a manner that helps prevent interference from online cognition.^41^

In control participants, replay-DMN coupling during rest was specifically boosted for replay events containing task-relevant (inferred) relationships (*β*_inferred_), from before to after learning. This is in line with reports that the content of offline reactivations is biased towards behaviourally salient (and recently learned) information, serving to prioritize memory consolidation and assimilation of new information into existing knowledge structures.^64-68^ Consistent with this hypothesis, we find that the strength of replay-DMN coupling after learning (and specific for inferred transitions) predicts subsequent explicit knowledge of abstracted features of the learned task structure (i.e. ordinal position of task pictures).

In patients, there was no similar increase in task-relevant replay-DMN coupling, and indeed this effect was negative. It is important to note that *β*_inferred_ reflects the predictive influence of task-relevant replay on DMN activation (i.e. replay events containing task pairs that are adjacent in a learned sequence), controlling for the mean coupling effect over all replay events (*β*_non-specific_). Thus, a drop in *β*_inferred_ from before to after learning does not reflect a reduced overall effect of replay-DMN coupling *per se* (captured by *β*_non-specific_, which is equivalent between groups) but rather a reduction in the specificity of this effect for task-relevant replay transitions. Given the proposed role of replay, we speculate that a reduction in this specificity might have deleterious effects on stabilization of task representations and memory. Our prior hypothesis was that such abnormalities in replay-DMN coupling might relate to symptoms such as hallucinations (given a proposed role of replay-DMN coupling in minimizing the interference between memory reactivations and goal-oriented cognition^41^) and maladaptive (delusional) beliefs (given a proposed role in belief updating). However, we find no relationship between positive psychotic symptoms and replay-DMN coupling. This might reflect a temporal fluctuation and context dependency of many core symptoms.

Most previous resting state studies of DMN in schizophrenia have availed of fMRI, and differ substantially from the HMM approach we use, both in operationalization of DMN activity (commonly defining this as the magnitude of blood oxygenation-level–dependent signal within voxels of a DMN mask) and the degree to which DMN dynamics are temporally resolved (many studies averaging DMN activation or connectivity over all samples within a rest session). This literature has yielded a wide array of (often equivocal) findings pertaining to group differences in functional connectivity between nodes of the DMN,^17,26,69^ functional connectivity between nodes of DMN and ‘task positive’ networks^13,17,26,70,71^ and graph-theoretic properties of whole-brain functional networks.^72^ Attenuated task-related DMN deactivation is among the most well-replicated fMRI findings in the condition.^19-27^ Nevertheless, a cohesive synthesis of this literature has remained elusive, owing in part to considerable methodological heterogeneity.^29,73^

By contrast to an established fMRI approach, the HMM approach to MEG resting state analysis operationalizes each RSN as a spectrally resolved pattern of power covariations and coherence distributed over brain regions, which is held fixed across all participants. This allows for an inference of individual participant RSN activation dynamics with millisecond resolution.^4,52^ Emerging evidence indicates that such information may uncover hitherto undetected differences in RSN dynamics in clinical samples,^74^ although to the best of our knowledge, the approach has not previously been applied to MEG or electroencephalography (EEG) data in schizophrenia. Previous studies have established that an HMM approach, as applied to MEG resting state data, identifies RSN patterns corresponding to those detected using fMRI.^2,4,41,75^ Our ascription of RSN2 as DMN, owing to high power in medial prefrontal regions and high coherence in parietal regions, is in line with these previous reports. Additional support for the interpretation of RSN2 as DMN comes from the strong association between this network and replay events (see Supplementary Fig. 3 and Higgins *et al.*^41^) (previously detected using fMRI^42^), as well as our finding of stimulus-evoked RSN2 deactivation during stimulus localizer, a deactivation that is attenuated in patients in a manner predicted by previous fMRI studies^19-27^ (Fig. 4).

With any case-control design, consideration needs to be given to whether neural differences might arise secondary to more general (non-specific) group differences in neural dynamics or cognition. Our demonstration of a group difference in replay-DMN coupling is not explained by differential expression of replay *per se* (as demonstrated empirically in the present paper) nor in a differential ability to detect task state reactivations (demonstrated in matched task decoder generalization accuracy between groups^47^). Likewise, the observed group difference is unlikely to be attributed to differences in the HMM fitting procedure between groups (including a differential concordance between the RSN state observation models and individual participant RSN features) as patients and controls had similar RSN spectral characteristics in the frequency range used for model fitting (1–45 Hz), exhibited similar dynamical properties of inferred RSN state time courses and similar baseline (non-specific) replay-evoked RSN activation profiles. Moreover, group differences in stimulus-evoked RSN profiles are unlikely to be driven by differential task engagement, given matched stimulus localizer task performance. Finally, we find no evidence that our neural effects are explained by antipsychotic exposure, although a strict test of this hypothesis requires a randomized placebo-controlled experimental design.

## Conclusions

We show schizophrenia is associated with a reduced resting state coupling between DMN activation and replay events. This coupling is specific for inferred task relationships and predicts subsequent memory maintenance for such relationships. The finding sheds light on previously reported resting state DMN abnormalities in schizophrenia and and raises further hypotheses that these might reflect neural processes relevant to memory consolidation and inference. Furthermore, replay-DMN coupling is positively related to stimulus-evoked DMN suppression, providing a point of contact to an established fMRI literature pertaining to task-related DMN modulation in schizophrenia Our findings thus provide a pointer towards how the computational function of offline RSN dynamics may be relevant for understanding the complex phenomenology of schizophrenia, opening up new avenues of investigation of resting state brain activity in psychiatry.

## Supplementary Material

fcad056_Supplementary_Data

## Data Availability

MATLAB code for TDLM (replay detection) available at: https://github.com/YunzheLiu/TDLM. MATLAB code for RSN time course inference and alignment to replay available at https://github.com/OHBA-analysis/Higgins2020_Neuron. See Supplemental Materials and Methods for data availability.

## Abbreviations

D2/3R: dopamine 2/3 receptor
DMN: default mode network
fMRI: functional MRI
HMM: hidden Markov model
MEG: magnetoencephalography
RSN: resting state network
SWR: sharp wave ripple
TDLM: temporally delayed linear modelling
