# Supplementary material for: Reduced coupling between offline neural replay events and default mode network activation in schizophrenia
Source: Brain Commun. 2023 Mar 3;5(2):fcad056. doi: 10.1093/braincomms/fcad056 (PMC10026370; doi:10.1093/braincomms/fcad056)
Supplement: fcad056_Supplementary_Data [file fcad056_supplementary_data.pdf]

Reduced coupling between offline neural replay events and default mode network activation in schizophrenia

Matthew M Nour, Yunzhe Liu, Cameron Higgins, Mark W Woolrich, and Raymond J Dolan

SUPPLEMENTARY MATERIALS AND METHODS

Datasets and participants

General exclusion criteria for Dataset A were anticonvulsant or benzodiazepine medication, age > 45 years, poor vision limiting performance and not having been educated in English. Healthy volunteers were not taking neurological or psychiatric medication, had no history of neurological or psychiatric disorder (assessed by SCID-I [1]), and no family history of psychosis. Patients had no neurological or psychiatric comorbidity.

Participants were classified as antipsychotic-free if they had been free from antipsychotic treatment for at least 6 weeks for oral or 6 months for depot formulations, consistent with previous approaches [2]. Patients were either taking antipsychotic medication (n = 15, see **Supplementary Table 1** for details) or not taking any medication at all. Medication was not stopped for this study for those patients taking medication. Healthy volunteers were not taking any medication.

We assessed psychiatric symptoms with the Positive and Negative Syndrome Scale (PANSS) scale [3], Montgomery Åsberg Depression Rating Scale (MADRS) [4], and General Assessment of Function (GAF) [5]. We administered brief measures of IQ (the Wechsler Test of Adult Reading, WTAR [6]) and working memory (mean of forward and backward Digit Span).

Applied learning task and MEG sessions (Dataset A)

The following task description is taken from our published report using the same dataset (Nour et al, (2021)), in which we focused on detection of neural replay and representational similarity structure [7]. Participants performed a validated task during MEG, where they inferred how 8 distinct task pictures were embedded within two separate 'structural sequences' ([A→B→C→D] & [A'→B'→C'→D']), without ever being shown either complete sequence order [8]. Instead, participants viewed scrambled 'visual sequences' containing pictures from both 'structural sequences'. During pre-scan training (visit 1) participants were explicitly

shown how 8 training pictures embedded within 3 'visual sequences' could be mapped onto 2 correct 'structural sequences'. Specifically, they were informed that in each 'visual sequence' (e.g., [C→D→C'→D']) only the first and last transitions correspond to correct 'structural relationships' (e.g., [C→D & C'→D'] are 'structural' transitions, but [D→C'] is a 'visual-only' transition), and their understanding was checked subsequently by asking them to explain this 'unscrambling rule'. Once this understanding was established, participants completed 3 Structure Learning sessions in which they practiced how to infer the correct structural relationships between the 8 training pictures; learning not only how the specific training pictures were associated, but also encoding a stimulus-independent mapping from 'visual' to 'structural' sequences (i.e., the 'unscrambling rule'). During MEG (visit 2) participants completed 3 similar Applied Learning sessions, with an entirely new picture set of 8 pictures, thus applying the encoded task schema to novel stimuli.

In both the pre-scan training session (visit 1), and MEG Applied Learning sessions (visit 2) participants were presented with a set of 3 unique 'visual sequences' (e.g., [C→D→C'→D'], [B'→C'→B→C], & [A→B→A'→B']), and this set was itself repeated 4 times (i.e., 12 sequence presentations). In each 'visual sequence' presentation the 4 pictures were presented sequentially from left to right, concurrent with a single word text descriptor at the bottom of the screen (each picture 175*175 pixels, presented for 1.5 s, with an inter-stimulus interval of 300 ms separating the first and last pair of pictures, and 1100 ms separating the second pair of pictures. Inter-sequence interval 3 s). The structural embedding of each picture within 'visual' (and, by extension, 'structural') sequences was counterbalanced between subjects.

Following each Applied Learning session, we assessed knowledge of structural sequences (i.e., the picture→state embeddings) with a 12-question quiz. For each question a single target picture (e.g., B) was presented on the center of the screen for 5 s, followed by the appearance of two probe pictures (e.g., A and D) at the bottom left and right of the screen. Participants indicated which of the two probe pictures *comes later* than the target picture in the 'structural sequence' with a button press, with no time restrictions or feedback. Laterality of the correct/incorrect pictures was randomly selected on each trial. Patient and control participants were matched in quiz performance at the end of the final Applied Learning session (immediately prior to a 5 minute post-learning rest session) and also immediately after this rest session. However, patients showed impaired knowledge of inferred sequences after the first and second Applied Learning session (a complete analysis of behavioral results from this dataset is presented in our prior published report, Nour et al, Cell (2021)[7]).

During MEG, prior to Applied Learning, participants completed a 5 minute rest session with eyes open (PRE, at the start of the scanning session). This was followed by a Stimulus Localizer task, which provided training data for the stimulus decoders required for sequenceness analysis. On each trial of the Stimulus Localizer participants were presented a task picture on the center of the screen (320*320 pixels, 1 s), followed immediately by presentation of a single-word text descriptor, and indicated whether the text matched (50% trials) or did not match (50% trials) the preceding picture with a button press (2 s response window, no feedback, laterality of yes/no response counterbalanced between subjects). Each picture was shown many times (mean 51.0, SD 3.48) in a randomized order. The inter-trial interval was drawn uniformly from 700 – 1300 ms on each trial. Following Applied Learning, participants completed a second 5 minute rest session with eyes open (POST), which ended with a 4th (post-rest) knowledge quiz to assess knowledge retention. At the end of the scan session participants completed a Position Probe task. This differed from the Stimulus Localizer task in one respect alone. Here, each picture was followed by a single *number* (1, 2, 3 or 4), and participants now indicated whether this number matched the *position* of the preceding picture with a button press (2 s response window, no feedback, chance accuracy 50%, laterality of yes/no response counterbalanced between subjects). Pictures were presented in randomized order.

The task was implemented in MATLAB (MathWorks) using Cogent (Wellcome Trust Centre for Neuroimaging, University College London) v 1.30.

MEG acquisition

For Dataset A, 3 sensors were not recorded due to excessive noise in routine testing, and the task was projected onto a screen suspended in front of participants. Participants responded using two buttons (L/R) of a MEG-compatible button box (Current Designs) held in the right hand. For Dataset B, MRI data (used for MEG coregistration) were acquired using a Philips Achieva 7T scanner.

MEG Data preprocessing

For the replay detection pipeline (Dataset A), we filtered sensor space data to a pass band of 0.5 to 50 Hz (including a notch filter for power line artefact at 48 – 52 Hz) and downsampled to 100 Hz. For the RSN analysis pipeline, we filtered sensor space data to a pass band of 1 to 45 Hz and downsampled data to 250 Hz. In both pipelines excessively noisy MEG data segments (rest data), trials (stimulus localizer data), and sensors were removed, followed

by automated detection and removal of artefactual MEG components identified using independent component analysis (ICA)

As outlined in our previous study [9], the use of a slightly amended filter pass band for RSN analysis (1 – 45 Hz) ensured RSN-state dynamics were not driven by low frequency sensor drift effects or mainline power noise effects, which the HMM modeling approach is more sensitive to compared to the replay identification methods. Similarly, the higher sampling rate used for RSN analysis pipeline (250 Hz) ensured sufficient resolution of the time embedding to enable good estimation of spectral content for each RSN-state definition.

For analyses of RSN activation dynamics we excluded epochs and trials that included MEG samples which had been marked 'bad' in the RSN analysis pipeline. There was no difference between patient and control participants in the proportion of excluded ('bad') samples in post-learning rest MEG data (controls mean = 6.31% ± 1.18, patients 7.34% ± 1.63, $z(55) = 0.09$, $P = 0.93$, Wilcoxon rank sum test, two tailed), number of excluded MEG sensors in post-learning rest MEG data (controls mean = 3.38 ± 1.10, patients = 4.04 ± 1.56, $z(55) = -0.99$, $P = 0.32$, Wilcoxon rank sum test, two tailed), nor in the number of excluded stimulus localizer trials (controls mean = 32.7 ± 3.55, patients = 44.0 ± 4.93, $z(55) = -1.781$, $P = 0.075$, Wilcoxon rank sum test, two tailed).

ICA (FastICA, http://research.ics.aalto.fi/ica/fastica) was used to decompose the sensor data for each session into 150 temporally independent components and associated sensor topographies. Artefact components (e.g., eye blink and mains interference) were classified by automated inspection of the spatial topography, time course, kurtosis of the time course and frequency spectrum for all components [8]. Artefacts were rejected by subtracting them out of the data.

Replay analysis pipeline

In our original report we used a Temporally Delayed Linear Modelling (TDLM) framework [10] to quantify extent to which correct (inferred) transitions between task states were expressed in state reactivation time courses (e.g., transition [A→B] is expected to manifest in spontaneous neural reactivation of state A, $\sigma(X\beta_A)$, reliably preceding that of state B, $\sigma(X\beta_B)$). We considered such sequential (time-lagged) reactivation patterns at different time lags, τ (corresponding to 'neural replay' of inferred structure at different replay speeds), and in a manner that controls for non-specific lagged reactivation patterns and the effect of a background alpha oscillation in MEG data [7]. We found evidence for significant replay of inferred structure in a predominantly forward direction, with maximal evidence (across the sample of patients and

controls) at τ = 40 ms lag [7], a lag corresponding also to findings from earlier reports in healthy volunteers [8,11].

In the present study we used this empirically identified replay lag (τ = 40 ms) to estimate a separate 'replay probability' time course for each unique task state transition (8 * 8 = 64), for each rest session and participant. For example, the replay probability time course corresponding to transition A→B ($R^{[A\rightarrow B]}$) was computed as the elementwise product of the reactivation time course of state A and time lagged reactivation time course of state B (i.e., $R_t^{[A\rightarrow B]} = A_t * B_{t+\tau}$). This probabilistic output was thresholded at the transition-specific 99th percentile [9] to provide the time course of 'replay onsets' used throughout this paper.

Source space reconstruction

The RSN analysis pipeline was conducted in source space. As in Higgins et al. (2021), Dataset B was co-registered to MRI structural information using a multiple local sphere forward model [12], while Dataset A, which did not have associated MRI, was co-registered using (nasion and pre-auricular) fiducial markers. Both Datasets then underwent the same source space reconstruction pipeline.

After co-registration we beamformed the broadband MEG signal (1 - 45 Hz) to source space (8mm MNI grid), using a linearly constrained minimum variance (LCMV) beamformer [13]. We then parcellated this grid into 38 regions of interest (ROIs) derived from an independent component analysis of fMRI resting state data from the Human Connectome Project [14], to derive a single activity time course per ROI (defined as the first principal component of the activity within the ROI voxels, with voxel contribution weighted by the anatomical parcellation). Following parcellation we performed a spatial leakage correction using a symmetric multivariate orthogonalization approach as outlined in [14,15]. This approach identifies the set of orthogonal ROI time courses that are least displaced from the original time courses, thus removing zero-lag correlations between ROI time courses. Finally, we applied a sign-flipping procedure to deal with the sign ambiguity inherent in beamforming (i.e., time courses of the same ROIs from different subjects/sessions have an arbitrary sign) [15,16], applying the sign flip 'template' used when processing Dataset B (used to fit RSN state observation models) [9] to the beamformed parcellated MEG data of all participants from Dataset A.

Resting state network modeling

We use an established hidden Markov model (HMM) approach to infer the activation time courses of each RSN state [15,16]. The HMM framework makes a distinction between a directly observed variable, X, and a discrete hidden (latent) variable, Z, which generates X. At any timepoint t, $(t \in [1,2,\ldots,T])$, a single latent state is active $(Z_t \in [1,2,\ldots,K])$. The relationship between each latent state and the observed data, X_t, is captured by a state-specific probabilistic 'observation model' (i.e., a state-conditional probability distribution $P(X_t|Z_t,\theta)$). The transition probability between discrete latent states is governed by a $[K,K]$ transition probability matrix, π, which respects the first-order Markov conditional independence property:

$$P(Z_t|Z_{t-1}) = P(Z_t|Z_{t-1}, Z_{t-2}, Z_{t-3}, \ldots, Z_1) = \pi \quad (1)$$

, where $\pi^{i,j} = P(Z_t = j|Z_{t-1} = i)$.

Thus, the full posterior probability of the general HMM model (i.e., joint probability of all data observations, $X_{1:T}$, inferred latent state time courses, $Z_{1:T}$ and inferred HMM parameters, $\Theta = \{\theta, \pi, \pi_0\}$ is given by:

$$P(X_{1:T}, Z_{1:T}, \Theta) = P(X_1|Z_1,\theta)P(Z_1|\pi_0)P(\pi_0)P(\theta)\prod_{t=2}^{T} P(X_t|Z_t,\theta)P(Z_t|Z_{t-1}, \pi)P(\pi)P(\theta) \quad (2)$$

, where $P(X_t|Z_t,\theta)$ specifies the observation model (parameterised by θ), $P(Z_t|Z_{t-1},\pi)$ species the latent state transition probabilities (parameterised by π), $P(Z_1|\pi_0)$ denotes the initial state probability (parameterised by π_0). The prior probability of the HMM parameters Θ, i.e., $P(\pi_0)$, $P(\pi)$, and $P(\theta)$ are chosen to be non-informative conjugate distributions [15,17,18].

A key feature of such models is that the observation model parameters (θ) are static with respect to time. As the observations, X_t, are conditional only on the active latent state (Z_t) and these observation model parameters (θ), an observation X_t is conditionally independent of all previous and future observations (X_i, where $i \neq t$) given Z_t. Thus, all time-varying dynamics in the observed data, X, are explained by the activation dynamics of the latent state variable, Z.

The form of the observation model, $P(X_t|Z_t,\theta)$ is a key analysis choice, and may be tailored to the data features best captured by the imaging modality and of most relevance to the

experimental question [15]. In the present work we use a HMM with Time Delay Embedding (HMM-TDE) approach, in which the observation model associated with each RSN state reflects the distribution of spectrally-resolved power at each ROI, and coherence between ROIs. This reflects an understanding that spectrally-resolved phase coupling between brain regions is a key mechanism of cross-region communication ('functional connectivity') in the brain [15,16]. The HMM-TDE observation model augments ('temporally embeds') the observation at each time point t with MEG data from $2l$ adjacent time points before and after t, as follows:

$$P(vec(X_{t-l:t+1})|Z_t = k) \sim N(0, \Sigma_k) \quad (3)$$

, where the vec operator performs the temporal embedding at each time point, t. Specifically, the temporally embedded MEG data at each time point t, $X_{t-l:t+1}$, corresponds to a vectorised $[W, P]$ data matrix, where P is the number of original source-space data features (e.g., ROIs), and W is the size of the temporal embedding window (in number of samples) centred on t (i.e., $W = 2l + 1$). This vectorisation thus augments the source-space MEG data, X, from dimensionality $[T, P]$ to dimensionality $[T, WP]$.

As outlined in Equation 3, the temporally embedded data at each time point, $vec(X_{t-l:t+1})$, is then modelled as a multivariate normal distribution with zero mean ($[WP, 1]$) and a state-specific covariance matrix, Σ_k ($[WP, WP]$). As the WP data features reflect the activity in adjacent time points across all ROIs, Σ_k reflects the RSN-specific autocovariance on each ROI (how the activity in a given region evolves over time as a function of its past activity, i.e., power) and the cross-covariance between ROIs (how the activity in a given region evolves over time as a function of the past activity in another region, i.e., coherence) [9,16].

The full HMM posterior in Equation 2 is amenable to variational Bayesian methods, a family of optimisation-based approaches to approximating a posterior distribution. This approach yields an estimate of the posterior probabilities pertaining to the (time-invariant) latent state observation models (θ), in addition to posterior estimates for parameters governing the dynamics of latent state activation in the data (e.g., $P(\pi_0)$ and $P(\pi)$), and the inferred RSN activation time courses themselves. These studies performed inference on the concatenated MEG timecourses across participants, to yield a fixed set of observation models across participants, thus facilitating comparison of inferred RSN activation dynamics across subjects [16,17].

A slightly amended approach to analysis of RSN activation dynamics is described in Higgins et al. (2021)[9]. In this study the authors first fitted the full HMM model to a reference dataset (Dataset B, comprising 5 minutes of MEG rest data from 55 healthy participants, each with high resolution MRI [16]) to yield a single set of RSN state observation models, and then applied the (now fixed) observation models to a new MEG dataset (which lacked high resolution structural MRI). This approach affords greater confidence and reproducibility with respect to the spatial distributions of activity in each RSN state observation model, owing to availability of high-resolution MRI used for MEG co-registration in Dataset B. In Higgins et al. (2021), model inference (in Dataset B) was run 5 times, using stochastic gradient variational Bayes methods to iteratively learn a full model thorough batch training. The model with the lowest free energy of these 5 runs was subsequently used for all analyses. K (number of RSN hidden states) was set to 12, as in [16]. As discussed in this earlier study, the choice of K influences the granularity of the resulting analysis (higher K resulting in RSN observation models at finer resolution). $K = 12$ was found to yield RSN observation models that had close correspondence to previously described RSNs in the fMRI literature (e.g., RSN2 = DMN).

In the present work we re-use the identical RSN observation models fitted by Higgins et al. (2021) (shown in **Supplementary Figure 1**) to infer RSN activation dynamics in the rest and localizer task MEG data from each participant in Dataset A separately, yielding participant-specific posterior estimates of the state-specific activation time courses and first order transition probabilities. This approach enables a direct comparison between our results and the previously published MEG results using the same observation models [9]. As in Higgins et al. (2021), we set $l = 9$ (36 ms) and work with observed data, X, not in the raw (temporally embedded) source space (feature dimensionality WP), but in a lower dimensional space capturing the axes of maximal variance (first 80 principal components of a PCA analysis on temporally embedded source-space data).

We adopt the RSN labelling (1, 2, …, 12) used in Higgins et al. (2021), where the numerical proximity of two states reflects the probability of observing a (bidirectional) transition between them, captured in the inferred one-step state transition matrix, π. As in this previous study, RSN 2 is the DMN. A similar data-driven RSN labelling procedure in our own data (Dataset A) yields a highly similar 1-dimensional state ordering (see **Supplementary Figure 2** for mean empirical transition matrices in patient and control samples).

Further discussion of epoching RSN activation dynamics by replay onsets

As described in main text, for each participant, rest-session, and replay transition, we identified timepoints where 'replay evidence' exceeded the 99th percentile (where replay evidence is defined as reactivation probability of 1st task picture multiplied by the lagged reactivation probability of 2nd task picture). We then used the identified timepoints (replay onsets) to epoch the RSN activation time course and calculated the mean RSN activation epoch for each replay transition in turn (n = 64). Prior to averaging the individual epochs for each transition, we first excluded epochs which contained (transition-specific) suprathreshold replay onsets prior to 0 ms. This ensured that we only included RSN activation epochs that contained an 'event-free baseline' (for the transition in question). Note that we had no similar restriction for time points after 0 ms (such that 0 ms could represent the first of multiple suprathreshold events). We did not include epochs where this event-free baseline overlapped with the previous (transition specific) epoch.

Further discussion of transition-specific epoching procedure

Our transition-specific epoching approach has two key advantages. First, it allows us to flexibly examine the replay-evoked [time, RSN state] dynamics corresponding to different hypotheses pertaining to task transition structure, as described in the main text. For example, to, to capture the replay-RSN coupling effect that is *specific* for inferred transitions ($\beta_{inferred}$), beyond any background effect, we regressed the [transition, 1] vector of RSN activations (for each time and RSN in turn) onto a design matrix comprising regressors both for inferred (structurally adjacent) transitions and all (non-specific) transitions (as in **Figure 3**, wherein we additionally calculate the change in such an effect from pre- to post-learning rest sessions, $\Delta\beta_{inferred}$. For this regression analysis we first downsampled the epoched RSN activation time course to 100 Hz for computational expediency). A second advantage of our approach is that by using transition-specific thresholding we ensure that our summary estimates of replay-RSN coupling over transitions are not unduly influenced by differences in output magnitude between different neural decoders (within participants).

Extracting primary axis of variation in evoked RSN dynamics

The primary approach we adopt to analyzing evoked RSN dynamics (illustrated in **Figure 3B**) is well suited to analyzing activation effects arising at individual time*RSN combinations. However, it is ill-suited to capturing the global patterns of evoked RSN dynamics

that span several time points and RSNs. Consequently, we use a complementary analysis, in which we project the [event, time*RSN] matrix of evoked RSN activations (events may be individual replay transitions or stimulus localizer trials, and where the [time, RSN] data corresponding to each event has been stacked into a single dimension) on to a principal component (PC) space, [event, PC], where each PC feature is defined as a [time, RSN] coefficient matrix capturing an axis of variation (between individual events) in the observed data.

Specifically, we use a Principal Component Analysis (PCA) to define the mapping between native and PC space, such that the 1st PC (used throughout this manuscript) represents the axis capturing maximal variance in the observed data ('principal axis of variation'). To ensure that our projection from native to PC space is identical for all participants, we conduct the PCA on the evoked neural data concatenated over all participants. Thus, in the case of replay-evoked RSN dynamics the PCA was performed on a [subjects*session*transitions, time*RSN] matrix, while in the case of the stimulus-evoked RSN dynamics the input data was [subjects*trial, time*RSN] (in both cases mean-centering columns of the input matrix prior to PCA, so that the resulting principal eigenvector captures the mode of principal variation between events). We conduct separate PCAs for replay-evoked and stimulus-evoked data reflecting our primary hypothesis that the principal axes of variation in the evoked neural response differs between these two conditions. This procedure allows us to extract, for each participant, a scalar 1st PC 'score' for each event, which captures with a single number the extent to which this 1st PC [time, RSN] coefficient pattern is expressed. For each participant, we can use these values to compute a mean stimulus-evoked RSN activation profile (mean 1st PC score over stimulus localizer trials) and the RSN activation profile uniquely associated with inferred replay transitions ($\beta_{inferred[PC]}$, derived from regressing the [transition, 1] vector of scores, from replay-evoked data, onto a design matrix of inferred transitions and a constant term).

Dynamical properties of replay onsets

We present an analysis of replay interval times, conditional on DMN activation, in **Supplementary Figure 3C**. For each participant and replay transition we first identified all suprathreshold replay onsets that coincided with time points in which the DMN was the maximally active state, and calculated the mean time between each of these onsets and the subsequent replay onset (interval time), excluding intervals containing artefactual MEG

samples [9]. For each participant we then averaged the DMN-conditional interval times over all replay transitions, and subtracted the mean interval time computed in the rest session as a whole (i.e., participant-specific baseline interval time) from this number.

Dynamical and spectral properties of RSN activation time courses

We present several descriptive analyses of inferred RSN dynamics in **Supplementary Figure 2**, computed for each participant separately. 'Fractional occupancy' describes the relative contribution of each RSN activation to the [time, RSN] activation time course in the rest session. 'Interval time' describes the mean time separating two suprathreshold RSN activations, and 'lifetime' describes the mean duration of an epoch of continuous suprathreshold RSN activations (in both cases threshold defined as 0.67, and computed such as to be unaffected by presence of artefactual MEG samples) [9]. To capture the predictability (non-uniformity) of RSN state transitions we calculate the entropy the inferred one-step Markovian transition matrix for each participant (excluding self-transitions on the diagonal and normalizing the remaining entries to sum to 1). The entropy of the resulting discrete probability distribution is defined as,

$$H(X) = -\sum_{i=1}^{n} P(x_i) lnP(x_i) \tag{4}$$

, where n is the total number of (non-self) one-step RSN state transitions, and $P(x_i)$ is the (normalized) transition probability corresponding to transition x_i.

Spectral profiles for each RSN were extracted separately for each participant by fitting a multitaper to the MEG data itself, conditioned on the active RSN (taper window length 2 s, frequency resolution 0.5 Hz, range 1 – 45 Hz, applying 7 Slepian tapers) [9,19]. This provided an empirical assessment of the power and coherence for each ROI as a function of frequency for each participant. We use the mean of these effects over all ROIs (power) and ROI pairs (coherence) to define a summary effect for RSN, for each participant, at each frequency **(Supplementary Figure 2E** & **F**).

Statistical analysis and software

For all analysis we defined outlier participants as those exhibiting effect sizes >3.5 SD ± group median. For replay-evoked MEG analysis, this corresponded to 2 control participants (2 for structure learning performance) and 1 patient (sequenceness effect size meeting outlier

criteria), identically to our original replay paper [7]. For analyses restricted to stimulus localizer sessions, where knowledge of task structure was irrelevant, we excluded 2 participants (1 patient, 1 control) for outlier behavioral performance during the concurrent attention task.

We used non-parametric permutation tests to assess the statistical significance of effects where we wished to control for multiple comparisons across time points (i.e., evoked RSN dynamics) or frequencies (i.e., RSN spectral properties). This procedure involved repeatedly computing the effect of interest at each time or frequency sample for each of 500 participant-level permutations where the nature of the permutation instantiated the relevant null hypothesis. To test for significant effects greater than 0 (i.e., one-sample hypotheses, e.g., replay-evoked DMN activation > 0 shown in **Figure 3B & 3C**) in each permutation we flipped the sign of each participant's effect time course with 50% probability (sign-flip permutation). To test for significant differences in these effects between patients and controls (i.e., two-sample hypotheses, as in **Figure 3D**) in each permutation we randomly assigned 27 participants as 'controls', and the remaining participants as 'patients' (group-membership permutation). For both one- and two-sample tests, for each permutation we then identified the maximal group-level effect of interest (for peak-level effects this corresponded to the maximal effect over all time or frequency samples, while for cluster-level this corresponded to the cluster exhibiting the greatest magnitude, as described below). The distribution of maximal effects over all permutations thus defined an empirical null distribution of effect sizes that controlled for multiple comparisons over samples. Any effect in the true (unpermuted) data that exceeded the 95th percentile of this distribution was deemed statistically significant at $P_{FWE} < 0.05$. We used a peak-level significance threshold for our primary analysis involving replay-evoked DMN activation for inferred transitions (**Figure 3**), reflecting the temporally-localized nature of these effects. For one-sample hypotheses the effect of interest was the mean DMN activation over participants (right-tailed hypothesis, **Figure 3B & C**), while for group-difference hypotheses we used the absolute t value derived from a two-sample t-test (two-tailed hypothesis, **Figure 3D**). We used an analogous cluster-level significance approach when assessing significance of effects that manifested over a broader range of adjacent time points or frequencies (i.e., non-specific replay- and stimulus-evoked RSN activation profiles in **Supplementary Figure 3A & 4A**, and RSN spectral profiles in **Supplementary Figure 2E & Supplementary Figure 2F**). As in the peak-level analysis, for each permutation we first computed the effect of interest at each sample (t value of one-sample t-test for right-sided hypotheses, **Supplementary Figure 3A & 4A**, and absolute t value of two-sample t-test for two-sided group difference hypotheses, **Supplementary Figure 2E & Supplementary Figure 2F**). Cluster-level inference then

proceeds by (1) defining stretches of contiguous samples each exhibiting absolute t values > 3 (cluster defining threshold), (2) calculating the sum of absolute t values within each cluster ('cluster magnitude'), and (3) defining an empirical null distribution using the maximal cluster magnitude from each permutation. Any cluster in the unpermuted data exhibiting a magnitude greater than the 99.58th percentile of this null distribution was deemed statistically significant (i.e., $P_{FWE} < 0.05$, Bonferroni corrected for multiple comparisons over RSN states). This procedure is similar to that taken in our previous studies [7,9].

When considering single variable effects or bivariate correlations, we conducted a formal test that the effects in question were sampled from a population with a normal distribution (Shapiro Wilk test) prior to using parametric tests (e.g., unpaired t-test, Pearson's correlation), and used non-parametric equivalent tests where this null hypothesis was rejected at $\alpha = 0.05$ (e.g., Wilcoxon rank sum test for equal medians, correlation and regression analyses conducted on rank ordered variables). For between-subjects multiple regression analyses, 'group' was effects coded (patients = -0.5, controls = +0.5), unless otherwise specified. For all analyses summary effects are reported as mean ± 1 standard error of the mean (SEM), and two-tailed $P < 0.05$ is deemed significant, unless otherwise specified.

Statistical analysis was performed using MATLAB (Mathworks) 2019a. MEG pre-processing and source reconstruction was performed using MATLAB in conjunction with functions from the Statistical Parametric Mapping 12 (SPM12, https://www.fil.ion.ucl.ac.uk/spm/software/spm12/) toolbox, FieldTrip (http://www.fieldtriptoolbox.org/), the OHBA Software Library (OSL, including OAT, https://ohba-analysis.github.io/osl-docs/) and FMRIB Software Library (FSL, https://fsl.fmrib.ox.ac.uk/fsl/fslwiki/). HMM-TDE modeling was implemented using OHBA analysis code (https://github.com/OHBA-analysis/Higgins2020_Neuron).

Data and Code availability

Summary data used to generate the findings of this study are available upon request to the Lead Contact. This paper uses two datasets. Individual participant MEG data from Dataset A is available subject to participant consent. Dataset B was collected and held by the MEG UK Partnership, and access to this data by request to https://meguk.ac.uk/contact.

MATLAB code for TDLM (replay detection) available at: https://github.com/YunzheLiu/TDLM. MATLAB code for RSN time course inference and alignment to replay available at https://github.com/OHBA-analysis/Higgins2020_Neuron.

SUPPLEMENTARY RESULTS

Number of replay onsets detected per transition and across groups

For each participant and session (e.g., rest), we defined 'replay onsets' as time points in the replay-probability time course that exhibited high (> 99th percentile [9]) evidence of (transition-specific) sequential task state reactivation, preceded by a pre-onset baseline of subthreshold replay evidence (see **Supplementary Materials and Methods**). Using this criterion, we detected (mean) 87.9 ± SEM 1.91 individual 'replay onsets' per replay transition, across participants. Importantly, there was no difference between patients and controls in the number of identified replay onsets for any transition type (n = 64 two sample t-tests, one for each replay transition type, all P > 0.05, two tailed).

Replay-DMN in pre and post-learning rest sessions separately

The effect of task-relevant replay events on DMN activation ($\beta_{inferred[PC]}$) was not significantly different from 0 in the patient sample in either PRE not POST rest sessions (pre-learning: mean = 0.027 ± 0.019, one sample t-test, P = 0.172, two tailed; post-learning: mean = -0.011 ± 0.021, one sample t-test, P = 0.118, two tailed). By contrast, control participants showed a positive effect after learning (pre-learning: mean = 0.003 ± 0.017, one sample t-test, P = 0.860, two tailed; post-learning: mean = 0.029 ± 0.014 , one sample t-test, P = 0.049, two tailed).

Coupling between RSN activation and non-specific task state replay

Our primary results use a multiple regression approach to estimate the replay-DMN coupling effect that is specific for task-relevant replay transitions (i.e., that which exists over and above any 'replay'-DMN coupling that might exist for replay of state transitions that do not correspond to a learned task structure). For completeness, here we present the 'non-specific' replay-RSN effect, estimated for each RSN separately. This effect is simply the replay-RSN effect averaged over all (n = 64) replay transitions, estimated for each participant, per-replay time point, and RSN separately. We consider this a 'non-specific' replay-RSN effect as it makes no distinction between RSN activation dynamics linked to replay of 'correctly inferred' transitions (i.e., those between adjacent states in the task 'structural sequences', e.g., [A→B]), and those linked to non-specific transitions that do not reflect the inferred structure (e.g., [A→C']).

In both patients and controls 'non-specific' replay onsets were associated with activation in DMN, parietal alpha, occipito-parietal, and frontal networks (**Supplementary Figure 3A**, as in [9]), with DMN activation at replay onset showing a greater activation compared to any other RSN (all $P < 0.001$, 11 separate Wilcoxon signed rank tests for equal median activation between DMN and remaining RSNs, sample combining patients and controls, two tailed). A direct comparison of replay-evoked DMN activation between patients and controls revealed no significant group differences at any peri-replay time point (uncorrected $P > 0.05$ for all time bins -500 to +500 ms, Wilcoxon rank sum test for equal medians between independent samples at each bin, two tailed).

As outlined in the main **Results**, this analysis approach necessitates separate statistical tests for each peri-replay time point and RSN, and lacks sensitivity to the global pattern of evoked RSN dynamics where this spans multiple time points and RSNs. Consequently, to capture such an ensemble response pattern for individual replay transitions and participants, we conducted a principal component analysis (PCA) on the concatenated RSN activation time courses from all participants ($n = 64$ observations per participant and rest session, corresponding to the mean ensemble response for each unique replay transition), and extracted the principal axis of variation in neural dynamics between transitions and participants (see **Materials and Methods** for details). The [time, RSN] coefficient values of the 1st principal component (1st PC, 'principal axis of variation') revealed a 'DMN activation' profile (**Supplementary Figure 3B**), indicating that this principal mode of variation (capturing maximal variance between transitions and participants) aligns closely with mean evoked RSN dynamics across participants (shown in **Supplementary Figure 3A**). This activation pattern was expressed equivalently in both patient and control participants (mean 1st PC scores in controls = -0.019 ± 0.12, patients = 0.078 ± 0.17, $z(52) = 0.12$, $P = 0.90$, two sample Wilcoxon rank sum test for equal medians, two tailed, effect for each participant defined as the mean 1st PC score over $n = 64$ replay transitions). Patient and control participants also exhibited a similar preponderance for individual replay onsets to occur in a temporally clustered manner during periods of DMN activation, compared to the same effect computed in the rest session as a whole (DMN-conditional interval time vs. session mean, control = -0.26 seconds ± 0.03, patient = -0.28 seconds ± 0.02, $t(52) = 0.68$, $P = 0.51$, two sample t-test, two-tailed, **Supplementary Figure 3C**).

SUPPLEMENTARY FIGURES

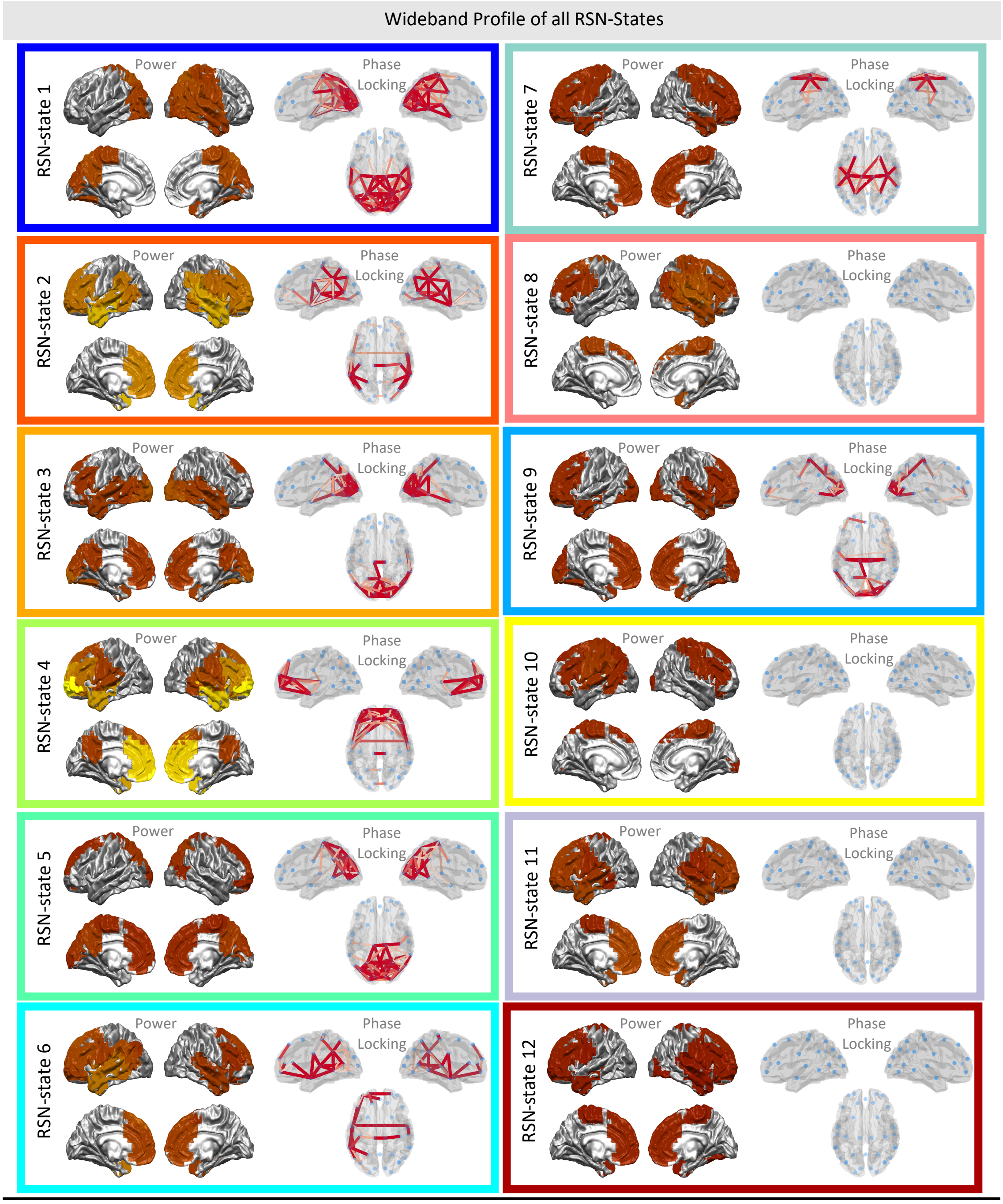

Supplementary Figure 1: RSN observation models. Related to Methods and Figure 2.
Spatial distribution of wideband power (left) and coherence (right) for each of the 12 RSN state observation models used for RSN time course inference in each participant and session. RSN2 is referred to as 'DMN' in this work. RSN models were fit to rest MEG data from Dataset B (n = 55), and are identical to the observation models used in Higgins et al. (2021) [9]. For each model we extracted spectral information at each of 38 ROIs by fitting a multitaper to the MEG data from Dataset B, conditioned on RSN activation probability (taper window length 2 s, frequency resolution 0.5 Hz, frequency range 1 – 45 Hz, 7 Slepian tapers) [9,19]. Wideband power plots are thresholded at 50% of the RSN-specific distribution. Coherence plots are thresholded using a Gaussian Mixture Model to identify significant edges within each RSN, as in [9]. Figure adapted from [9].

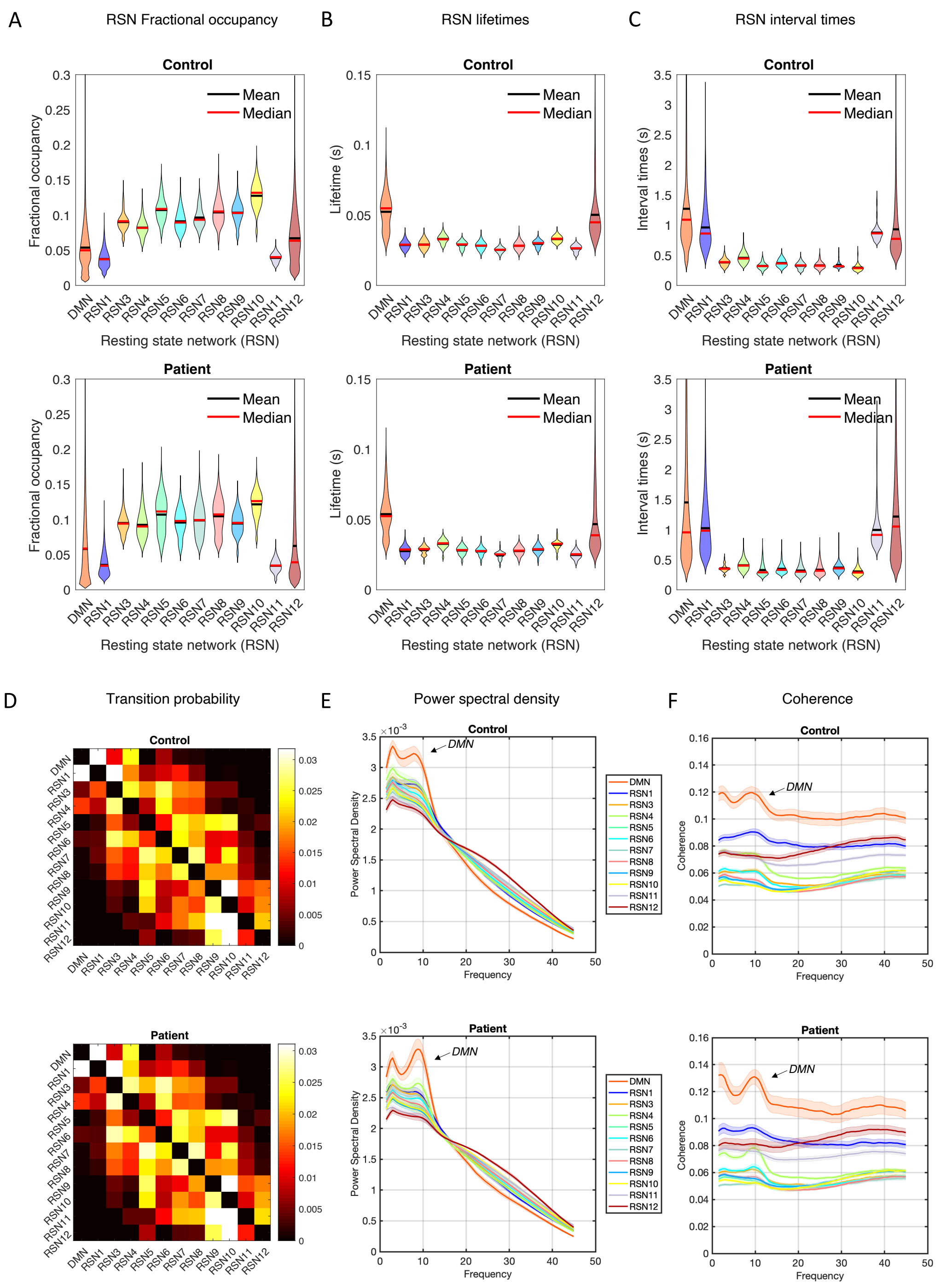

Supplementary Figure 2. Group comparisons in dynamical and spectral properties of inferred resting state network (RSN) timeseries. Related to Methods and Figure 2.

(A) RSN fractional occupancy (proportional activation of each RSN during rest). No significant difference between groups in any RSN (Wilcoxon rank sum test for equal medians at each RSN, Bonferroni corrected for multiple comparisons across RSNs).

(B) RSN lifetimes (mean duration of continuous RSN activation, not including epochs that span an artefactual 'bad' MEG sample). No significant difference between groups in any RSN (Wilcoxon rank sum test for equal medians at each RSN, Bonferroni corrected for multiple comparisons across RSNs).

(C) RSN interval times (mean duration separating two non-contiguous activation periods for the same RSN, not including intervals that span an artefactual 'bad' MEG sample). No significant difference between groups in any RSN (Wilcoxon rank sum test for equal medians at each RSN, Bonferroni corrected for multiple comparisons across RSNs).

(D) RSN empirical transition matrices inferred from HMM fitting procedure for each participant separately. The finding that numerically adjacent states exhibit elevated transition probabilities is expected as the numerical assignment of each state is based on the empirical transition matrix derived from the model fitting procedure on Dataset B, as previously described [9]. There was no group difference in the entropy of the RSN transition probability distribution (a measure of the uniformity of RSN state 1-step transition patterns, excluding self-transitions. Controls = 22.86 ± 0.12, patients = 22.67 ± 0.12, $t(52) = 1.43$, $P = 0.16$, two sample t-test, two tailed).
(E) Mean ± SEM (over subjects) power spectral density (PSD, averaged over ROIs) for each state. For each participant we extracted RSN-specific PSD at each frequency by fitting a multitaper to the MEG data, conditioned on RSN activation probability (taper window length 2 s, frequency resolution 0.5 Hz, frequency range 1 – 45 Hz, 7 Slepian tapers) [9,19]. For each RSN state, we tested for frequency windows (clusters) exhibiting a significant PSD difference between patients and controls using a non-parametric group-membership permutation test (500 permutations, cluster magnitude defined as the sum of absolute t values within a frequency window in which the effect size of the absolute group difference exceeds $t = 3$, see **Supplementary Materials and Methods**). We found no clusters exhibiting a significant group difference in any RSN at $P_{FWE} < 0.05$ (Bonferroni corrected for multiple comparisons over RSN states).
(F) Mean ± SEM (over subjects) coherence (averaged over unique ROI-pairs) for each state. Analysis and significance testing as in in (E).
Analyses (A) – (C) are calculated using the thresholded probabilistic activation time series for each RSN (threshold = 0.67). Violin plots generated using gaussian kernel density. All analyses are from post-learning rest MEG data. Sample: n = 27 controls, n = 27 patients.

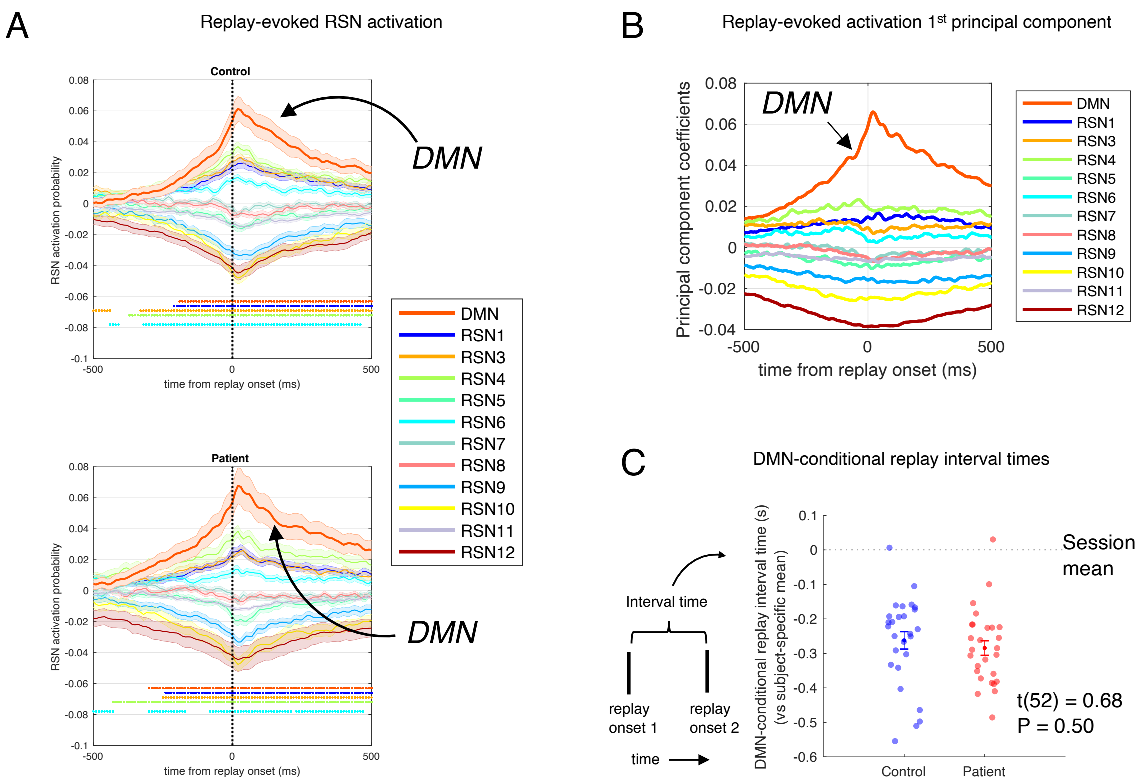

Supplementary Figure 3. Resting state network activation coincident with 'non-specific' replay of task states.

(A) Mean ± SEM (over participants) of RSN state activation dynamics at time points around replay onsets during post-learning rest (mean 'non-specific' effect across all possible state→state transitions, n = 64). There is a significant temporal association between replay onset and DMN activation (top: controls, bottom: patients). Coloured * below activation time courses represent time windows exhibiting a significant activation (above 0, session mean) at $P < 0.05$, family-wise error (FWE) corrected over time at the cluster level, and Bonferroni corrected over states, using a non-parametric sign flip permutation test (see **Supplementary Materials and Methods**) [9].

(B) Coefficients of the 1st principal component (1st PC) of replay-evoked RSN dynamics, capturing the principal axis of variation between replay events and participants (from a PCA on replay-evoked RSN activation time courses, concatenated over subjects, see **Supplementary Materials and Methods** for details. Identical to that shown in **Figure 3**). For visualisation, the [time*RSN, 1] vector of 1st PC coefficients has been reshaped back to the [time, RSN] matrix dimensions of the replay-evoked RSN activation time courses.

(C) Mean ± SEM (over participants) of the DMN-conditional replay interval times, defined as the mean temporal separation between two consecutive suprathreshold replay onsets, when the first onset occurs when the DMN is the maximally active RSN state. Single-participant effects presented as the difference between the observed DMN-conditional interval time and the participant-specific session mean replay interval time (negative values denote greater temporal clustering of replay onsets during DMN activation compared to session mean). Summary effects for each participant computed as the mean over all 64 replay transition types. All analyses conducted in post-learning rest. N = 27 controls and n = 27 patients.

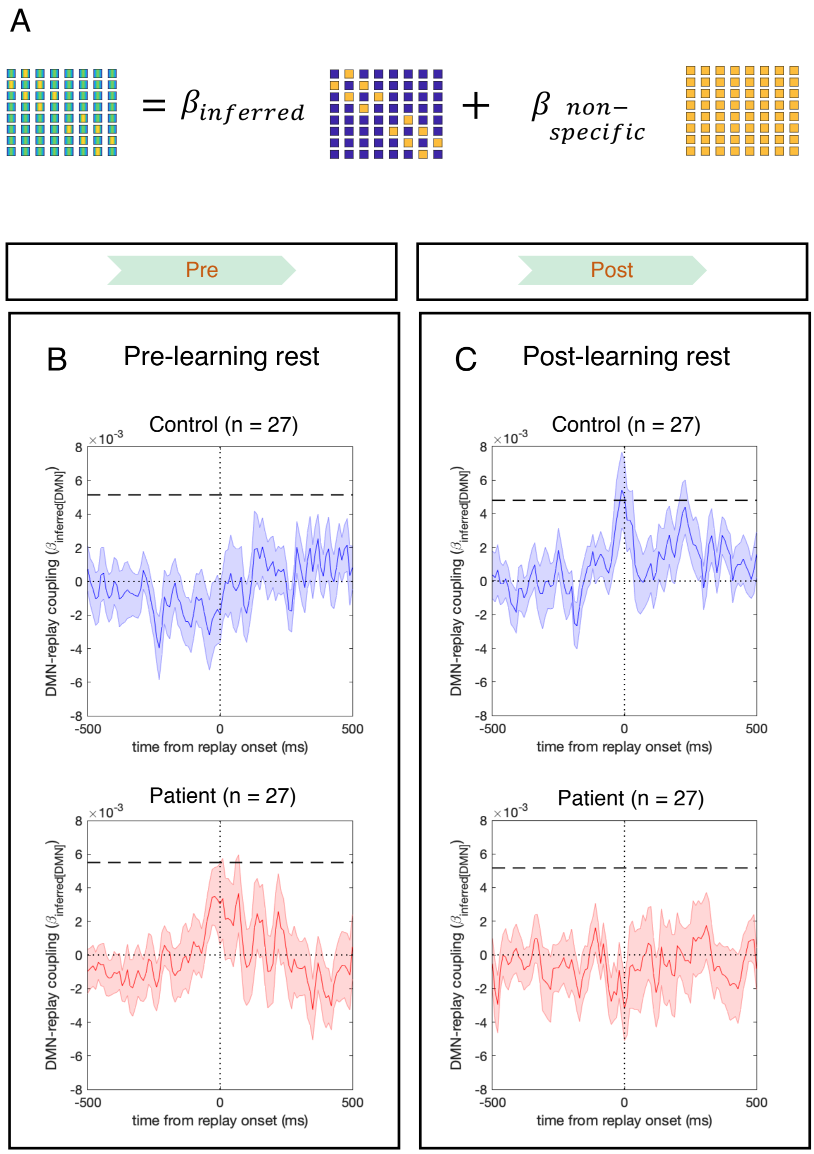

Supplementary Figure 4. Replay-DMN coupling, specific for inferred transitions, before and after learning. Related to Figure 3.
(A) Multiple regression approach to extracting the replay-DMN coupling effect specific for inferred transitions ($\beta_{inferred[DMN]}$), as shown in **Figure 3**.
(B) Mean ± SEM (over participants) $\beta_{inferred[DMN]}$ effect around replay onset, measured during pre-learning rest. No time point exhibits an effect exceeding a peak-level $P_{FWE} < 0.05$ in control (top) or patient (bottom) participants ($P_{FWE} = 0.05$ right-tailed statistical threshold depicted by horizontal dashed line, derived from non-parametric sign-flip permutation test, temporal region of interest -100 to +100 ms, 500 permutations).
(C) Mean ± SEM $\beta_{inferred[DMN]}$ effect measured during post-learning rest. Control participants (top) exhibit significant replay-DMN coupling at replay onset at peak-level $P_{FWE} < 0.05$. Significance threshold as in (B).

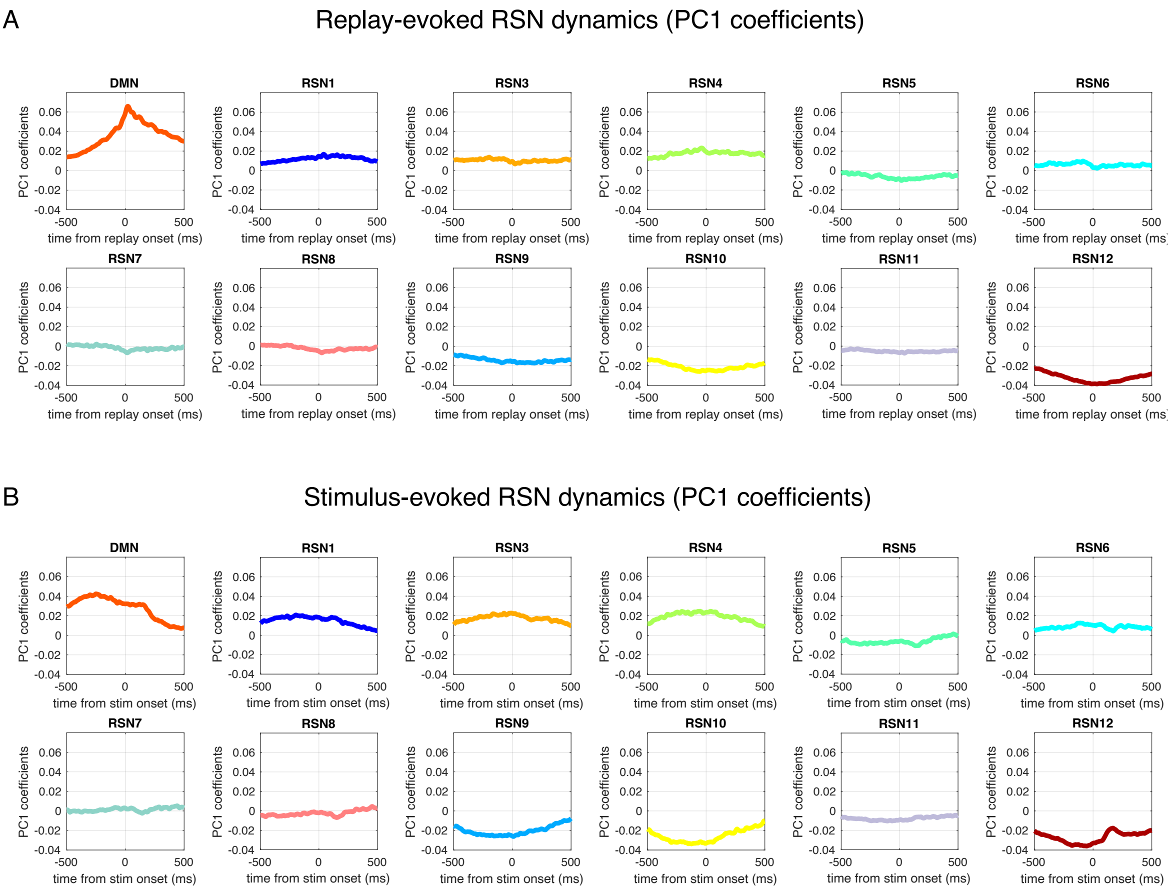

Supplementary Figure 5. RSN activation dynamics for each RSN separately. Related to Figures 3, 4,5.
(A) Coefficients of the 1st PC of the replay-evoked RSN activation time courses.
(B) Coefficients of the 1st PC of the stimulus-evoked RSN activation time courses.
In both cases, the PCA was performed on the [time, RSN] activation data, concatenated over all subjects and sessions (see **Supplementary Materials and Methods).**

SUPPLEMENTARY TABLES

Variable	Healthy volunteers	Patients	Group comparison[#]
**Demographic**			
Sample size	29	28	
Gender	6 F, 23 M	6 F, 22 M	x^2 = 0.005 (P = 0.95)
Age (mean, SD)	28.1 (6.53)	28.2 (5.26)	t = -0.08 (P = 0.93)
Years in education (mean, SD)	18.1 (3.5)	17.0 (3.2)	t = 1.28 (P = 0.21)
Employment status [F/P/U]*	7 / 8 / 14	10 / 3 / 15	x^2 = 2.72 (P = 0.24)
Handedness	25R, 4L	26R, 2L	x^2 = 0.67 (P = 0.41)
Ethnicity [W / BAME / Other]†	10 / 13 / 6	10 / 15 / 3	x^2 = 1.13 (P = 0.57)
Alcohol units week^{-1} (mean, SD)	3.86 (5.51)	3.18 (6.00)	t = 0.45 (P = 0.66)
Current recreational cannabis (not within 1 week)	9	11	x^2 = 0.43 (P = 0.51)
Current smoker (not within 6 hours)	6	13	x^2 = 4.25 (P = 0.04)
**Cognitive**			
IQ (SD)	103.3 (6.15)	103.9 (8.43)	t = -0.30 (P = 0.77)
Digit span forward (mean, SD)	6.33 (0.96)	6.16 (1.18)	t = 0.59 (P = 0. 56)
Digit span backward (mean, SD)	4.16 (0.99)	3.73 (1.16)	t = 1.51 (P = 0.14)
**Psychiatric symptoms and signs**			
Depressive symptoms‡ (mean, SD)	0.89 (2.53)	8.71 (5.58)	t = -6.66 (P < 0.001)
Positive psychotic symptoms§ (mean, SD)	7.14 (0.35)	14.4 (6.21)	t = -6.29 (P < 0.001)
Negative psychotic symptoms§ (mean, SD)	7.07 (0.26)	14.4 (6.56)	t = 6.013 (P < 0.001)
General psychopathology§ (mean, SD)	16.4 (0.98)	25.4 (7.08)	t = -6.74 (P < 0.001)
General assessment of functioning‖ (mean, SD)	98.0 (5.3)	69.4 (14.2)	t = 10.1 (P < 0.001)
**Clinical Details**			
Number taking D2/3R antagonist medication	-	15¶	-
Chlorpromazine eq. mg day^{-1} in medicated patients (median, IQR)[20]		66.7 (51.6)	
Months since first psychotic episode (median, IQR)	-	48 (30)	-
Number acute psychotic episodes (median, IQR)	-	3 (2)	-
Number inpatient admissions (median, IQR)	-	1 (3)	-

Supplementary Table 1. Participant demographic, cognitive and clinical information. Related to Materials and Methods.

* F = fulltime employment, P = part-time employment, U = unemployed (including student).

† W = White. BAME = Black, Asian, and Minority Ethnic. Other includes multiple ethnic groups.

‡ Montgomery Åsberg Depression Rating Scale (MADRS)[4], floor = 0.

§ Positive and Negative Syndrome Scale (PANSS) scale[3], floor = 7(positive), 7(negative), 16(general).

‖ General Assessment of Functioning (GAF) scored from 0 – 100.

¶ D2/3 antagonist medication per medicated patient: (1) olanzapine 15 mg day^{-1}, (2) olanzapine 10 mg day^{-1}, (3) lurasidone 18.5 mg day^{-1}, (4) aripiprazole 10mg day^{-1}, (5) lurasidone 37 mg day^{-1}, (6) risperidone 3 mg day^{-1}, (7) aripiprazole 400mg month^{-1} (depot), (8) risperidone 0.5 mg day^{-1}, (9) aripiprazole 5 mg day^{-1}, (10) olanzapine 7.5 mg day^{-1}, (11) olanzapine 10 mg day^{-1}, (12) amisulpride 400 mg day^{-1} & aripiprazole 5 mg day^{-1}, (13) paliperidone 50 mg month^{-1} (depot), (14) paliperidone 175 mg 3-month^{-1} (depot), (15) paliperidone 50 mg month^{-1} (depot).

#Group comparisons: unpaired t-test for continuous variables (two-tailed), Chi squared test for categorical variables (two-tailed).

SD: standard deviation. IQR: inter-quartile range.

Variable	Replay-evoked RSN dynamics ($\beta_{inferred[PC]}$, post learning rest)	↑ Replay-evoked RSN dynamics ($\Delta\beta_{inferred[PC]}$, 'post minus pre' rest)	Stimulus-evoked RSN dynamics (stimulus localizer)
Positive psychotic symptoms[3]	rho(25) = -0.04, P = 0.76	rho(25) = 0.14, P = 0.48	rho(25) = 0.17, P = 0.39
Negative psychotic symptoms[3]	rho(25) = -0.40, P = 0.04	rho(25) = -0.07, P = 0.74	rho(25) = 0.48, P = 0.01
Depressive symptoms[4]	rho(25) = -0.10, P = 0.62	rho(25) = -0.15, P = 0.45	rho(25) = -0.05, P = 0.79
D2/3R antagonist medication	t(25) = -1.64, P = 0.11	t(25) = -1.40, P = 0.17	t(25) = 1.86, P = 0.08

Supplementary Table 2. Relationship between evoked RSN dynamics and clinical variables. Related to Figure 5.
Correlations performed with Spearman's correlation (owing to non-normality of clinical variable distribution). Medication effect tested with two sample two tailed t-test (owing to normality of neural effects). Sample, n = 27 (one patient participant excluded from both analyses for fulfilling outlier criteria, see **Supplementary Materials and Methods**).

SUPPLEMENTARY REFERENCES

1. First MB, Spitzer RL, Gibbon M, Williams JBW. *Structured Clinical Interview for DSM-IV Axis I Disorders— Patient Edition*. Version 2. New York Biometrics Research Department; 1995.

2. Jauhar S, Veronese M, Nour MM, et al. Determinants of treatment response in first-episode psychosis: an 18F-DOPA PET study. *Molecular Psychiatry*. 2019;24(10):1502-1512. doi:10.1038/s41380-018-0042-4

3. Kay SR, Fiszbein A OLa. The Positive and Negative Syndrome Scale (PANSS) for schizophrenia. *Schizophr Bull*. 1987;13(2):261-276. doi:10.1093/schbul/13.2.261

4. Williams JBW, Kobak KA. Development and reliability of a structured interview guide for the Montgomery-Åsberg Depression Rating Scale (SIGMA). *British Journal of Psychiatry*. 2008;192(1):52-58. doi:10.1192/bjp.bp.106.032532

5. American Psychiatric Association. *Diagnostic and Statistical Manual of Mental Disorders*. 5th ed. American Psychiatric Publishing; 2013.

6. Wechsler D. *Wechsler Test of Adult Reading: WTAR*. The Psychological Corporation; 2001.

7. Nour MM, Liu Y, Arumuham A, Kurth-Nelson Z, Dolan RJ. Impaired neural replay of inferred relationships in schizophrenia. *Cell*. 2021;184(16):4315-4328. doi:10.1016/j.cell.2021.06.012

8. Liu Y, Dolan RJ, Kurth-Nelson Z, Behrens TEJ. Human Replay Spontaneously Reorganizes Experience. *Cell*. Published online 2019:1-13. doi:10.1016/j.cell.2019.06.012

9. Higgins C, Liu Y, Vidaurre D, et al. Replay bursts in humans coincide with activation of the default mode and parietal alpha networks. *Neuron*. 2021;109(5):882-893. doi:10.1016/j.neuron.2020.12.007

10. Liu Y, Dolan RJ, Higgins C, et al. Temporally delayed linear modelling (TDLM) measures replay in both animals and humans. *eLife*. 2021;10:e66917. doi:10.1101/2020.04.30.066407

11. Kurth-Nelson Z, Economides M, Dolan RJ, Dayan P. Fast Sequences of Non-spatial State Representations in Humans. *Neuron*. 2016;91(1):194-204. doi:10.1016/j.neuron.2016.05.028

12. Huang MX, Mosher JC, Leahy RM. A sensor-weighted overlapping-sphere head model and exhaustive head model comparison for. *Physics in Medicine & Biology*. 1999;44:423-440.

13. Veen BD Van, Drongelen W Van, Yuchtman M, Suzuki A. Localization of brain electrical activity via linearly constrained minimum variance spatial filtering. IEEE Transactions on. *Biomedical Engineering*. 1997;44(9):867-880.

14. Colclough GL, Brookes MJ, Smith SM, Woolrich MW. A symmetric multivariate leakage correction for MEG connectomes. *NeuroImage*. 2015;117:439-448. doi:10.1016/j.neuroimage.2015.03.071

15. Quinn AJ, Vidaurre D, Abeysuriya R, Becker R, Nobre AC, Woolrich MW. Task-evoked dynamic network analysis through Hidden Markov Modeling. *Frontiers in Neuroscience*. 2018;12(AUG):1-17. doi:10.3389/fnins.2018.00603

16. Vidaurre D, Hunt LT, Quinn AJ, et al. Spontaneous cortical activity transiently organises into frequency specific phase-coupling networks. *Nature Communications*. Published online 2018. doi:10.1038/s41467-018-05316-z

17. Baker AP, Brookes MJ, Rezek IA, et al. Fast transient networks in spontaneous human brain activity. *eLife*. 2014;2014(3):1-18. doi:10.7554/eLife.01867

18. Higgins C. Uncovering temporal structure in neural data with statistical machine learning models. *Thesis*. 2019;(WP-).

19. Vidaurre D, Quinn AJ, Baker AP, Dupret D, Tejero-Cantero A, Woolrich MW. Spectrally resolved fast transient brain states in electrophysiological data. *NeuroImage*. 2016;126:81-95. doi:10.1016/j.neuroimage.2015.11.047

20. David Taylor, Thomas Barnes, Allan Young. *The Maudsley Prescribing Guidelines*. 14th ed. Wiley-Blackwell; 2021.
